# DNA‐Guided Robust Single‐Protein Electronic Readout

**DOI:** 10.1002/advs.202516711

**Published:** 2025-12-17

**Authors:** Ziyi Ju, Zhaoxiang Deng, Yueqi Li, Jinghong Li

**Affiliations:** ^1^ Hefei National Research Center for Physical Sciences at the Microscale University of Science and Technology of China Hefei 230026 China; ^2^ Department of Chemistry School of Chemistry and Materials Science University of Science and Technology of China Hefei 230026 China; ^3^ Department of Chemistry Key Lab of Bioorganic Phosphorus Chemistry and Chemical Biology Tsinghua University Beijing 100084 China

**Keywords:** biosensing, DNA origami, protein conductance measurement, single‐molecular conductance

## Abstract

Electronic transport properties of single proteins offer powerful insights into their structure and dynamics. However, the orientation variability of electrode‐attached protein molecules poses a big difficulty in achieving precise electric measurements and reliable data interpretation. Here, a DNA origami‐based method that promotes preferred orientations of target proteins is reported, enabling more reproducible single‐molecule electrical characterization. With two orthogonal DNA aptamers installed inside a DNA origami nanocavity, a thrombin protein can be captured with a prescribed orientation between a gold substrate and a conductive AFM tip. Matrix‐patterned *I–V* measurements reveal that bivalently anchored thrombin molecules exhibit significantly reduced conductance variability compared to randomly adsorbed ones. This critical progress then enables the detection of a subtle conductance change of thrombin upon binding with Na^+^ or an inhibitor molecule. The generalizability of this approach is showcased by further applying it to a streptavidin protein. Moreover, the platform allows for the selective recruitment and electrical readout of proteins from a mixed sample, demonstrating the feasibility of single‐entity measurements within a complex molecular environment. This work provides a versatile platform for protein‐electrode interfacing with deterministic molecular orientation control, highlighting the potential of DNA nanotechnology in single‐protein electronic measurements.

## Graphical abstract

Electronic measurements of single proteins can reveal fundamental insights into their structure, dynamics, and interactions, but reproducibility is severely limited by the intrinsic structural heterogeneity of proteins combined with uncontrolled molecular orientation and electrode contact geometries. This work addresses the long‐standing challenge of achieving precise, label‐free, and reproducible single‐protein conductance measurements by introducing a DNA‐origami‐based platform that promotes preferred orientations of individual proteins for high‐confidence electrical characterization.

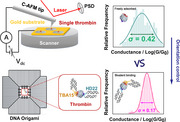

## 1 Introduction

Electronic properties of single protein molecules provide key insights into their structure, dynamics, and function.^[^ ^1^, ^2^, ^3^, ^4^, ^5^, ^6^ ^]^ However, the structural complexity and heterogeneity of proteins present major challenges for reproducible and interpretable electric measurements that rely heavily on electrode‐molecule contact.^[^ ^7^, ^8^ ^]^ A key source of variability is the random orientations and binding geometries of proteins at the electrode interface, which obscure the relationship between an electronic signal and a molecular structure. Addressing this challenge requires a strategy to reliably “mount” protein molecules with a defined orientation on the electrodes. To this end, several strategies have been developed previously. These include Au─S bonding via pristine or artificially engineered cysteine residues, which is often limited by the number and positions of available thiol moieties. In some cases, cysteine mutations may alter the native biochemical properties and charge transport characteristics of the protein.^[^ ^9^ ^]^ Alternatively, incorporating binding pairs like biotin‐streptavidin allows orientation‐specific protein‐electrode coupling but requires substantial structure modification of proteins.^[^ ^10^ ^]^ Electrostatically guided protein adsorption provides a facile, noncovalent way to build the protein‐electrode complex with limited interfacial conductance due to indirect protein‐electrode contact, which is only suitable for proteins with defined charge distributions.^[^ ^11^ ^]^ These pioneering efforts underscore the ongoing challenge and need for broadly applicable approaches that stabilize protein orientation without compromising structure or function.

DNA nanotechnology uses Watson–Crick base‐pairing to program the assembly of DNA and DNA‐conjugated objects into prescribed structures with nanometer precision. In particular, DNA origami is one such technology capable of making DNA structures with virtually arbitrary shapes and high spatial addressability ^[^ ^12^, ^13^, ^14^, ^15^ ^]^ by folding a long, circular, single‐stranded virus genome with the help of hundreds of short staple DNA strands. On the other hand, aptamers, evolved through systematic in vitro base‐sequence selection, can bind strongly with diverse protein targets.^[^ ^16^ ^]^ These two techniques together offer an unprecedented chance to place proteins in pre‐defined positions with high accuracy.^[^ ^17^, ^18^, ^19^ ^]^ In addition to aptamer‐mediated capture, proteins can also be directly bound to DNA‐origami scaffolds, providing an alternative approach for spatial positioning.^[^ ^20^, ^21^, ^22^, ^23^, ^24^ ^]^ In this work, we utilize aptamer‐functionalized DNA origami to control the orientation of single protein molecules sandwiched between a gold substrate electrode and a conductive atomic force microscopy (C‐AFM) probe. Building on a previously reported square‐shaped DNA origami nanocavity,^[^ ^25^ ^]^ we appended 20 thiols for stable Au─S bonding with the gold substrate. Two orthogonal thrombin aptamers are equipped on the two adjacent inner edges of the electrode‐exposing DNA nanocavity. The resulting DNA nanomodules, upon capturing protein molecules, are compatible with matrix‐patterned current‐voltage (*I–V*) measurements, enabling direct imaging and statistical conductance analysis of individual protein junctions.

By comparing among thrombin molecules with randomly oriented, monovalently tethered, and bivalently confined configurations, the bivalently fixated ones are found to exhibit markedly reduced conductance variability with a well‐defined Gaussian distribution, indicating improved orientation uniformity and electrode‐coupling stability. This optimal configuration further allows us to correlate as‐measured subtle conductance shifts with Na^+^‐ and D‐Phe‐Pro‐Arg‐chloromethylketone (PPACK)‐induced structural changes to thrombin, respectively, which cannot be achieved confidently for randomly adsorbed proteins. The versatility of this approach is demonstrated by successfully applying it to streptavidin, for which four identical aptamers are immobilized on the periphery of the DNA nanocavity to realize tetravalent protein binding. Moreover, benefiting from the accurate protein recognition by DNA aptamers and the improved signal confidence due to orientational protein/electrode coupling, the platform enables single‐molecule conductance measurements of target proteins directly from a mixed sample. This research establishes a general strategy for promoting orientation‐specific protein‐electrode coupling, highlighting the potential of DNA nanotechnology in high‐precision single‐molecule electronic measurements.

## 2 Results and Discussion

### 2.1 Assembly of DNA Origami Structures and Single‐Molecule Thrombin Binding

**Figure 1:**
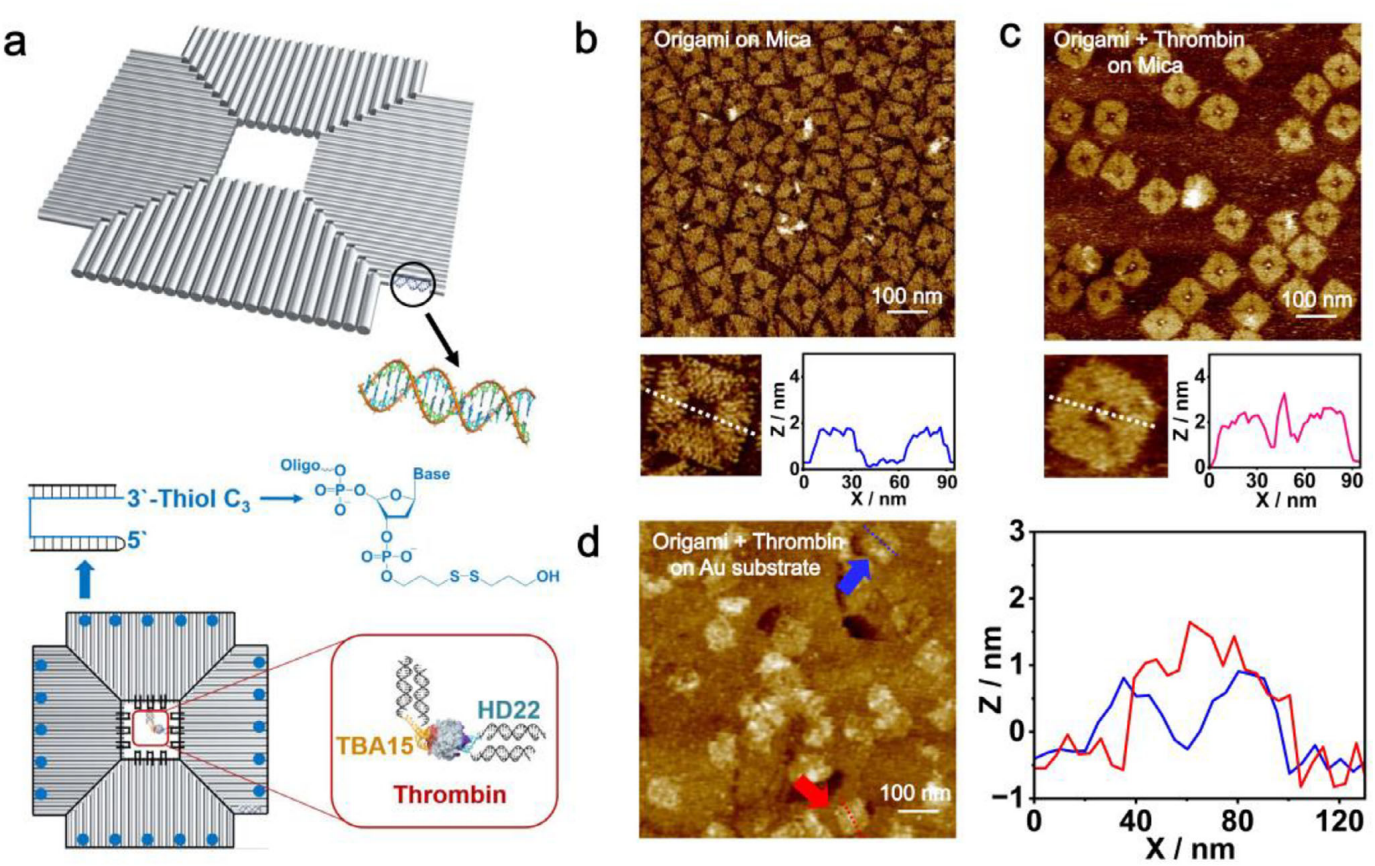
Design and characterization of DNA origami nanostructures for site‐specific thrombin binding. a) Molecular model of the DNA origami‐thrombin complex. The zoomed‐in schematic highlights the central cavity of the origami structure, where thrombin binds via two double‐stranded DNA stems capped with thrombin‐specific aptamers: HD22 (green) and TBA15 (orange). Blue dots indicate the positions of thiol‐modified staple strands, which are uniformly distributed along the edges for gold surface anchoring. b) AFM image of DNA origami structures deposited on mica under imaging buffer. c) AFM image of DNA origami incubated with thrombin under the same conditions. Zoomed‐in views in b and c show individual nanostructures; corresponding height profiles across their centers confirm the presence of central cavities and thrombin binding. d) AFM image of thrombin‐bound DNA origami structures adsorbed on a gold substrate and imaged in air. The overlaid height profiles compare origami with (red) and without (blue) thrombin, confirming successful protein binding and gold surface attachment.

To stabilize the spatial presentation of single proteins on electrode surfaces, we constructed square‐shaped DNA‐origami nanocavities, each functionalized with two aptamers to enable bivalent capturing thrombin inside the nanocavities. The resulting DNA/protein complexes were deposited on mica and Au (111) substrates and characterized by regular and conductive AFM measurements. Thrombin was selected as the target protein due to the availability of well‐characterized aptamers.^[^ ^26^ ^]^ The core design of the DNA origami was based on previously reports,^[^ ^25^, ^27^ ^]^ utilizing a single‐stranded M13mp18 virus genome (7249 bases) along with 203 short staple strands (23–57 bases) to build a square‐shaped DNA platform (80 × 80 × 2 nm^3^) with a central cavity (20 × 20 nm^2^) via scaffolded DNA assembly (**Figure** **1**a). Two DNA helices functionalized with thrombin‐binding aptamers (TBA15 and HD22)^[^ ^28^, ^29^ ^]^ extend into the cavity from perpendicular in‐plane directions, promoting binding at defined epitopes and thereby biasing the protein toward preferred orientations (Figure S1, Supporting Information). To enhance the deposition of the DNA origami on a gold (Au) electrode, the staple strands located at the outer edges (five on each side) of the DNA squares were purposely replaced by thiolated (disulfide) ones, enabling Au─S bonding between the origami and the electrode (Figure 1a). Detailed procedures for the construction of the DNA nanostructures can be found in the Experimental Section.

We first employed atomic force microscopy (AFM) to image the DNA nanostructures lacking thrombin‐binding aptamers on mica under an imaging buffer solution. AFM images verified the formation of square‐shaped DNA structures with central cavities (Figure 1b), consistent with the design. The DNA structures were found to be ≈80 nm in edge length and 2 nm in height under AFM, matching expected values.^[^ ^25^ ^]^ Upon incorporation of thrombin‐binding aptamers and incubation with thrombin proteins, over half of the origami structures displayed a central protrusion inside the cavity where the aptamers existed (Figure 1c). The height of the central feature (≈3.5 nm) is consistent with the known dimensions of thrombin,^[^ ^30^ ^]^ indicating that individual proteins were successfully captured by the aptamer‐functionalized origami.

To immobilize the thrombin‐bound DNA origami on a conductive substrate, we deposited the sample on a gold electrode, followed by the removal of excess solution. While the increased surface roughness of the gold electrode and its lower origami‐binding affinity compared to mica caused a significant reduction in image sharpness (Figure 1d; Figure S2, Supporting Information), the square‐shaped features bearing central cavities remained discernible. These results verified that the disulfide‐modified, thrombin‐loaded DNA origami structures were successfully anchored on the Au electrode.

### 2.2 Matrix Patterned C‐AFM Measurements of the Origami‐Thrombin Assembly

**Figure 2:**
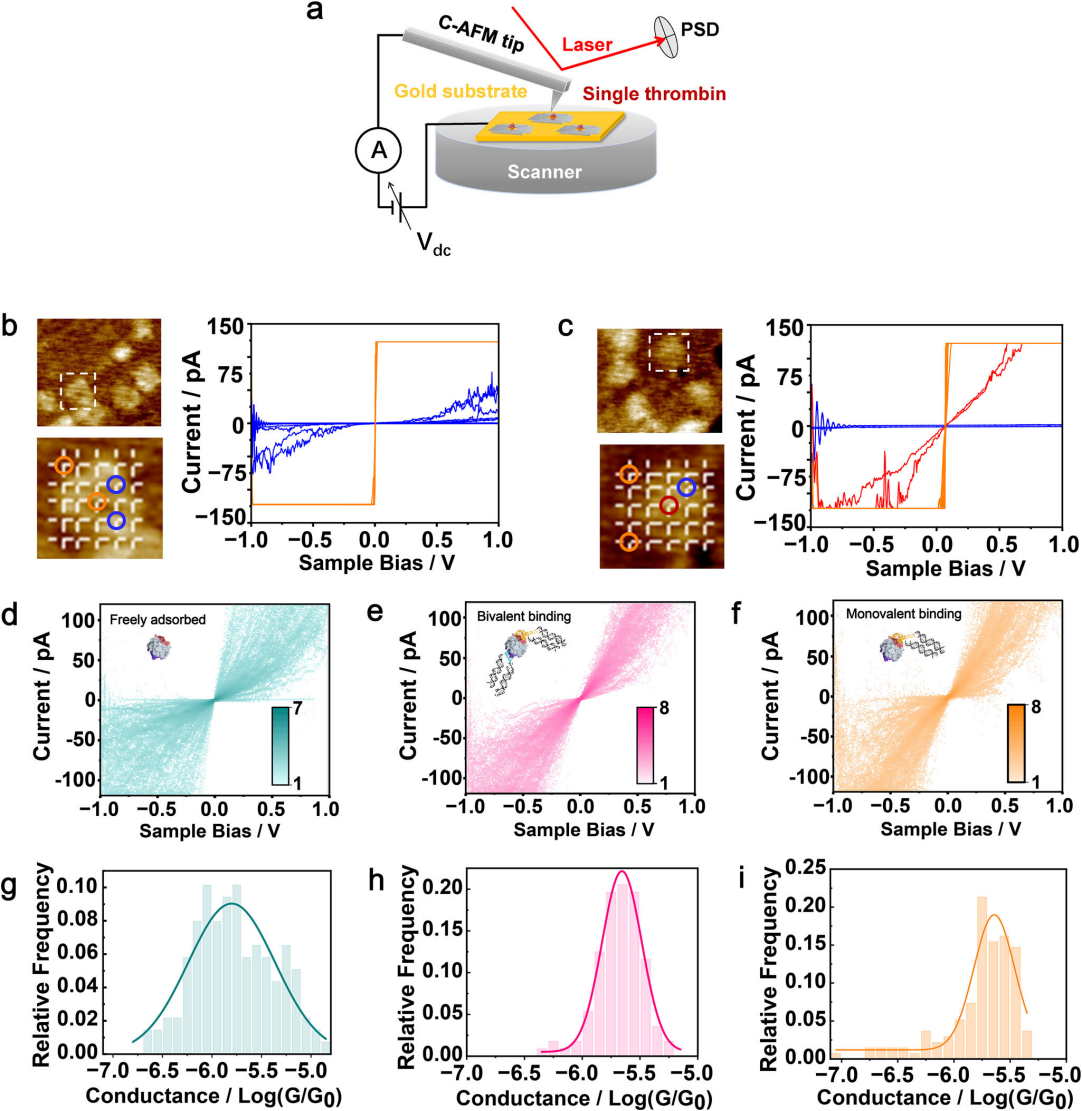
Measurement setup and *I–V* characteristics of thrombin‐origami assemblies. a) Schematic illustration of conductive AFM (C‐AFM) measuring a single thrombin molecule site‐specifically oriented by DNA origami. b) Matrix‐patterned *I–V* measurements taken at positions indicated by the cross‐points of the white grid (inset). Orange and blue curves are representative *I–V* curves taken on the Au substrate (orange circle) and DNA origami (blue), respectively. c) *I–V* curves from thrombin‐bound origami. The red curve corresponds to *I–V* measured at the central position of the array (red circle), representing the signal from a single thrombin molecule confined in the origami cavity. d–f) The intensity map of *I–V* curves recorded from: d) thrombin nonspecifically adsorbed on Au; e) thrombin bivalently anchored to DNA origami via two aptamers; f) thrombin monovalently anchored via a single TBA15 aptamer. g–i) Corresponding 1D conductance histograms from linear fitting within ±0.4 V for g) unanchored, h) dual‐aptamer anchored, and i) single‐aptamer anchored thrombin. The color bar represents the number of counts (density) in each grid. Both voltage (x) and current (y) axes are divided into 151 grids.

Following the attachment of the DNA‐origami‐confined single thrombin molecules to the Au surface, the resulting DNA/protein adducts were electrically characterized by conductive atomic force microscopy (C‐AFM) (**Figure** **2**a). After capturing an image of the origami‐modified Au surface (Figures S2 and S3, Supporting Information) with a Pt‐Ir coated AFM tip, imaging was paused, and a square frame slightly larger than the origami was selected. A 5 × 5 grid of evenly spaced sampling points was automatically defined within this frame, and current‐voltage (*I–V*) measurements (±1 V, 2 V s^−1^; 232 pN force) were then recorded to map the conductance response across the origami region.

In the case of the thrombin‐free origami (Figure 2b), the C‐AFM currents were below 50 pA at ±0.5 V when the tip was positioned over the DNA layers (blue curves). Such a current response was very weak. Statistical analysis of 66 measurements yielded an average conductance of 10^−7.18^ G_0_ (Figure S4 (Supporting Information), from linear fitting of *I–V* curves within ±0.4 V, G_0_ = 2e^2^/h ≈ 77.5 µS), consistent with previous experimental and theoretical investigations indicating that DNA origami exhibits low cross‐plane conductance.^[^ ^31^ ^]^ This low conductance is attributed to the overlapped phosphate backbones at crossover regions, which result in a reduced local density of states (DOS).^[^ ^32^ ^]^ In contrast, measurements taken on the Au surface outside the origami or within the central cavity of the origami showed significantly higher currents (orange curves). These currents exceeded the measurable limit (≈150 pA) of the C‐AFM module at a voltage bias of just a few millivolts, consistent with the conductive nature of Au.

For origami structures carrying thrombin proteins (Figure 2c), the current responses remained low over the origami body (blue curves) and high on the Au surface (orange curves). However, when the tip was positioned at the center of the origami—where thrombin was expected—a moderate current signal (red curves) was observed, much higher than that of the surrounding DNA origami but lower than that of the bare Au surface. This intermediate conductance is attributed to single thrombin molecules trapped in the nanocavities.

Control measurements on origami bearing aptamers but without thrombin showed cavity currents comparable to bare substrate (Figure S5, Supporting Information), consistent with the short, flexible aptamer stems residing near the cavity walls. These results suggest minimal contribution from protein‐aptamer dissociation.

Due to the relatively large tip size (radius of curvature ≈25 nm), the spatial resolution in C‐AFM mode was insufficient to accurately target the central thrombin molecules. Also, lateral tip drift during scanning might also occur. Nonetheless, separate high‐resolution imaging (performed on the same sample with a 2 nm probe) was first used to verify the successful assembly and deposition of the protein‐bound origami. Subsequently, matrix‐patterned *I–V* mapping with the C‐AFM tip enabled indirect localization of the central protein based on the characteristic higher‐conductance spot relative to the surrounding DNA regions. We anticipate that using sharper C‐AFM tips will further improve spatial resolution in protein localization and reduce conductance measurement uncertainty.^[^ ^33^ ^]^

To exclude possible collapse of proteins or substrate interference during I–V measurements, we performed force‐indentation control experiments using a high‐resolution probe (Figure S6, Supporting Information). The threshold forces required to deform thrombin and streptavidin significantly exceed the loading force applied in our electrical measurements, indicating that the proteins remain intact and properly positioned. In addition, tunneling contributions to the Au substrate at this separation are negligible, supporting that the measured signals originate from the protein‐electrode junction.

### 2.3 Conductance Profiling of DNA‐Origami‐Oriented Thrombin Molecules

To evaluate the molecular orientation effect on the electrical characterization of single proteins, we performed over 100 matrix‐patterned *I–V* measurements on individual thrombin objects and conducted statistical analysis. We first examined thrombin freely adsorbed on Au without DNA origami guidance. *I–V* measurements were performed on protein particles within partially exposed Au regions, and an intensity map of 138 *I–V* curves reveals a broad distribution, heterogeneous molecule‐electrode coupling, and random protein orientation (Figure 2d). To further assess single‐molecule conductance, we performed scanning tunneling microscopy break‐junction experiments,^[^ ^6^, ^34^ ^]^ which show conductance plateaus but with a broad distribution and no distinct histogram peak (Figure S7, Supporting Information), indicating substantial uncertainty for thrombin without controlled orientation. To extract conductance values from the *I–V* measurements, we linearly fitted each *I–V* curve within ±0.4 V to exclude oscillatory current points at higher biases. The resulting 1D histogram (Figure 2g) shows a broadened conductance peak centered at 10^−5.8^ G_0_ (σ = 0.42, R^2^ = 0.908), consistent with previous reports on proteins^[^ ^35^ ^]^ and small‐molecule junctions.^[^ ^36^, ^37^, ^38^ ^]^ Such a broad conductance distribution reflects uncertainty in both the number of molecules being probed and a variation in thrombin adsorption orientations.

Next, we characterized thrombin molecules oriented by DNA origami via site‐specific dual‐aptamer anchoring from perpendicular directions. The intensity map of 112 *I–V* curves (Figure 2e) displays an apparently more concentrated distribution, indicative of greater uniformity in transport behavior. A corresponding 1D histogram (Figure 2h) shows a narrower Gaussian peak centered at 10^−5.66^ G_0_ (σ = 0.17, R^2^ = 0.988), slightly higher in conductance than the freely adsorbed thrombin, but more importantly, with significantly reduced variability. This result suggests that the DNA‐origami‐based orientation strategy is able to generate more reproducible electrode‐protein interfaces via single‐molecule confinement. It is worth noting that, even in the bivalent binding case, thrombin conductance still exhibits a distribution with a standard error of ≈0.17 orders of magnitude. We attribute this remaining variability to small residual freedom in tilt and position, interactions with surface and origami, and intrinsic conformational dynamics of the protein. In addition, the random orientation of the DNA origami (facing up or down) may introduce moderate heterogeneity and broaden the conductance distribution, although this effect appears limited based on the rectification ratios (0.89 and 1.18) being close to 1(Figure S8, Supporting Information).

To further dissect the role of the DNA‐based protein orientation, we measured thrombin molecules anchored by only a TBA 15 aptamer (monovalent binding). The intensity map histogram of 136 *I–V* curves (Figure 2f) also shows a relatively narrow distribution, with the 1D conductance histogram (Figure 2i) yielding a peak at 10^−5.64^ G_0_ (σ = 0.18, R^2^ = 0.897). While the as‐measured conductance and its distribution width are similar to those of bivalently anchored thrombin, the slightly higher fitting error might suggest a slightly increased variability in thrombin orientation. This trend is consistent with the narrower AFM height distribution observed for bivalent binding (Figure S9, Supporting Information), suggesting a more stable and uniform spatial presentation of the protein. These results underscore the effectiveness of our DNA‐guiding strategy in orientational protein‐electrode coupling, thereby improving the precision of nanoscale bioelectronic measurements.

### 2.4 Detecting Na^+^‐Induced Structural Changes in Single Thrombin Molecules

**Figure 3:**
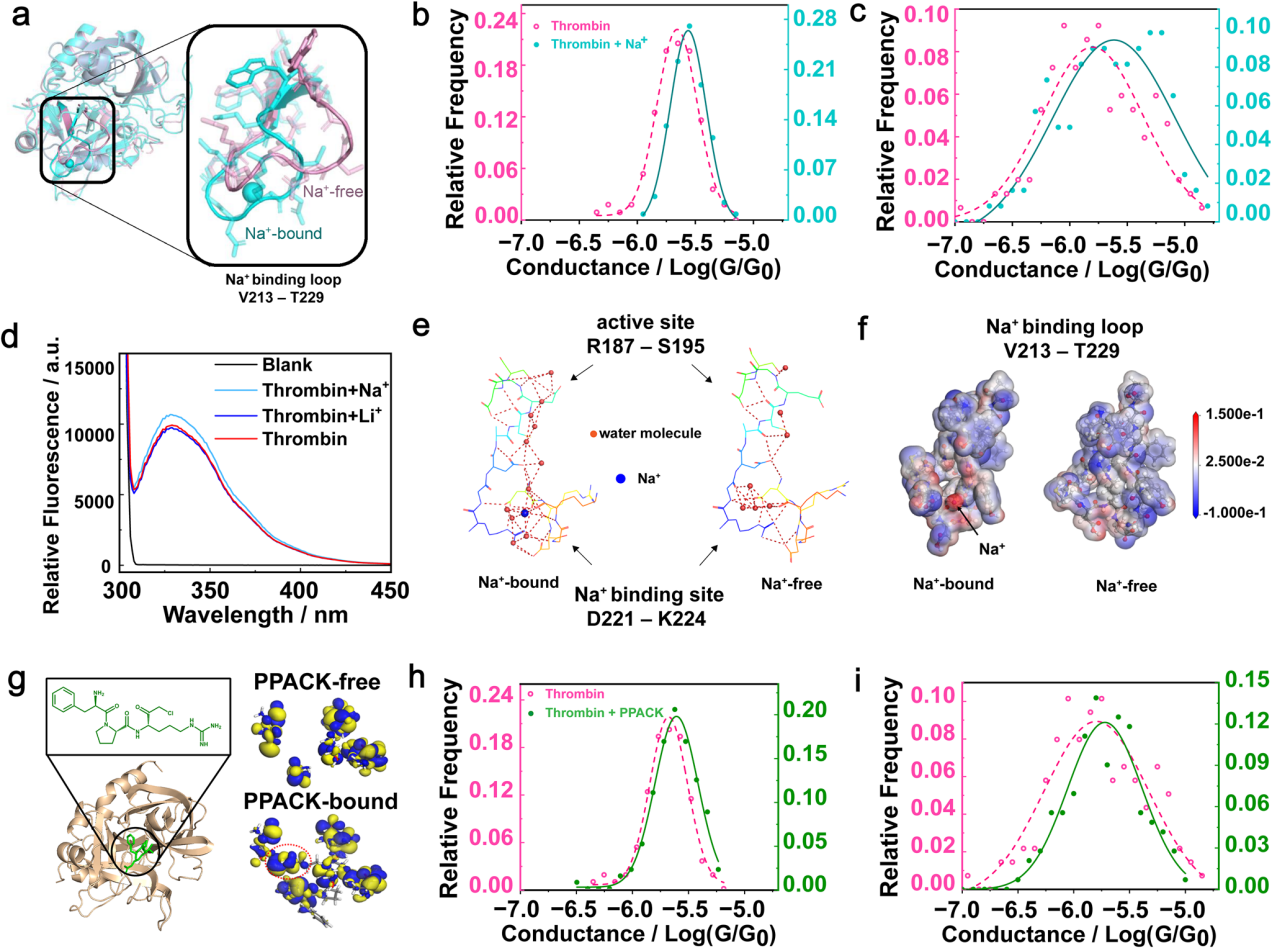
Ligand binding alters the conductance of single thrombin molecules. a) Structural comparison between the Na^+^‐bound (cyan, PDB: 3LU9) and Na^+^‐free (pink, PDB: 3BEI) forms of thrombin, highlighting conformational changes primarily in the Na^+^ binding loop (V213–T229). b) Conductance histograms of bivalently anchored thrombin molecules in the presence (cyan) and absence (pink) of Na^+^, showing a significant conductance increase (T = −26.62) upon Na^+^ binding with a narrow distribution. c) Conductance histograms of freely absorbed thrombin molecules under the same conditions as in b, exhibiting a broader distribution and reduced statistical robustness (T = −6.31). d) Fluorescence emission spectra (excitation at 280 nm) of thrombin samples (20 µL, 18.5 µM) recorded at 37 °C in buffer with 140 mM NaCl (light blue), 140 mM LiCl (dark blue), no added extra salt (red), and buffer alone (black). The binding of Na^+^ to thrombin enhances the fluorescence intensity without a change in λ_max_, while Li^+^ does not affect the fluorescence intensity or wavelength. e) Water network structure in the Na^+^ binding and active sites of thrombin. Red spheres represent water molecules, and the blue spheres represent Na^+^ ions. Structures drawn by Pymol. f) Calculated electrostatic potential distribution of the Na^+^ binding loop (V213–T229) in thrombin. The arrow indicates the position of the bound Na^+^ ion. g) Chemical structure of the thrombin inhibitor PPACK and its binding conformation in the thrombin catalytic site (left, PDB: 1PPB). Structures drawn by Pymol. Calculated molecular orbitals (right) sum from HOMO−5 to LUMO+5 for thrombin active site fragments without PPACK and inhibited by PPACK. h) Conductance histograms of bivalently anchored thrombin molecules with (cyan) and without (pink) PPACK, showing a conductance increase (T = −15.16) and slightly broadened distribution upon PPACK binding. i) Conductance histograms of freely absorbed thrombin molecules with and without PPACK, confirming the increase but with reduced confidence (T = −4.13) compared to the bivalently anchored configuration.

Having demonstrated the improved electronic measurements for the properly aligned thrombin molecules in the DNA origami nanocavities, we went further to explore this method for the detection of subtle structural changes in proteins. Previous spectroscopic, biochemical, and structural studies have shown that Na^+^ specifically interacts (K_d_ = 14 ± 1 mM)^[^ ^39^ ^]^ with thrombin to induce a minor conformational change, notably opening the enzyme's catalytic pocket, which enhances its catalytic efficiency by creating new binding sites (**Figure** **3**a).^[^ ^40^, ^41^, ^42^, ^43^ ^]^ To probe this event at the single‐molecule level, we performed conductance measurements on Na^+^‐ exposed thrombin‐origami complexes (Figure S10a, Supporting Information). Note that this sample had been incubated in a NaCl‐containing buffer (140 mM NaCl, 40 mM Tris‐acetic acid, 1 mM EDTA, 12.5 mM MgCl_2,_ 5 mM KCl). Gaussian fitting of the C‐AFM data revealed a conductance peak at 10^−5.56^ G_0_ (σ = 0.16, R^2^ = 0.986; 122 *I–V* curves, Figure 3b), indicating a higher conductance than that achieved in the absence of Na^+^ binding, while maintaining a narrow conductance distribution. For comparison, we also measured thrombin molecules randomly attached to the Au surface without the help of DNA origami upon binding Na^+^ (Figure S10b, Supporting Information). An average conductance of 10^−5.61^ G_0_ was found for this sample (σ = 0.53, R^2^ = 0.851; 136 curves, Figure 3c), which is also higher than that of the Na^+^‐free counterpart, but with a much broader distribution. While both the randomly deposited and bivalently anchored thrombin molecules showed increased average conductance upon Na^+^ binding, the latter produced a more statistically robust difference. Dual‐sample *t*‐tests (null hypothesis: the averaged conductance has no significant shift) yielded t‐values of −26.62 (bivalently anchored, *P* = 0.00018) and −6.31 (freely absorbed, *P* = 0.00247), confirming a much higher statistical significance for the orientation‐confined thrombin. These results demonstrate that the DNA‐origami‐based orientation control enhances the sensitivity and reliability of single‐protein electrical measurements to reflect subtle conformational changes.

To confirm that the conductance change was due to specific Na^+^ binding rather than nonspecific ion effects, we repeated the measurements by substituting NaCl with LiCl (140 mM), which interacts with thrombin nonspecifically (K_d_ = 250 ± 20 mM) and does not alter its structure.^[^ ^39^, ^44^ ^]^ The as‐measured average conductance was 10^−5.67^ G_0_ (σ = 0.19, R^2^ = 0.907, 72 curves, Figure S11, Supporting Information), comparable to the Na^+^‐free case. This result supports that the observed conductance increase originates from Na^+^‐induced specific structural changes in the protein.

The increased conductance of thrombin upon Na^+^ binding may arise from several factors. 1) Rearrangement of aromatic residues—binding of Na^+^ induces conformational changes in key aromatic residues (e.g., Y89, F227, W215), potentially enhancing their alignment with the charge transport pathway. The rearrangement of aromatic residues is supported by an increase in fluorescence emission of thrombin upon Na^+^ binding (see Experimental Section for details)^[^ ^41^, ^45^ ^]^ and reported XRD (X‐ray diffraction) structures (Figure 3d; Figure S12, Supporting Information).^[^ ^46^ ^]^ 2) Enhanced hydrogen bonding network—Na^+^ binding stabilizes a more extensive hydrogen bonding network involving 11 water molecules, compared to only 7 in the Na^+^‐free state, likely due to repositioning of E192 (Figure 3e).^[^ ^46^ ^]^ Hydrogen‐bond‐assisted conductance enhancement has been previously reported in single‐molecule studies.^[^ ^47^ ^]^ 3) Charge neutralization—our DFT (density functional theory) calculation reveals that Na^+^ can neutralize a negatively charged region involving residues D189, R221, K224, and the W215–E217 segment (Figure 3f), which could further facilitate charge transport (See Experimental Section for calculation details).^[^ ^48^, ^49^ ^]^

### 2.5 Probing Conformational Changes in Thrombin Upon PPACK Binding

We next investigated the conductance change of thrombin in response to an inhibitor, PPACK (D‐Phe‐Pro‐Arg‐chloromethylketone), which binds to thrombin in a 1:1 stoichiometry with high affinity (K_d_ ≈ 0.24 nM) (Figure 3g). Structural studies by NMR have shown that the thrombin‐PPACK complex closely resembles a transition‐state conformation during catalysis.^[^ ^50^ ^]^ Therefore, understanding the electronic properties of the protein‐inhibitor complex would offer insight into the conformational change of thrombin associated with its catalytic function. Previous NMR and accelerated molecular dynamics simulations suggest that the thrombin‐PPACK complex retains a dynamic structure,^[^ ^51^ ^]^ implying that it may still have an aptamer‐binding capacity and thus be compatible with the DNA‐origami‐enabled protein orientation strategy.

Thrombin (1.85 µM) was then allowed to incubate with PPACK (2 µM), and the resulting sample was diluted 10 times before interacting with the aptamer‐installed DNA origami. AFM imaging and an enzymatic activity assay confirmed the protein capturing by the DNA origami and the formation of the thrombin‐PPACK complex (Figure S13, Supporting Information). Matrix‐patterned *I–V* measurements were then performed to obtain conductance data of single thrombin‐PPACK complexes (Figure S14a, Supporting Information). As shown in Figure 3e, the conductance is centered at 10^−5.58^ G_0_ (σ = 0.20, R^2^ = 0.992, 134 curves), which is slightly higher (with a marginally broader distribution) than that of pristine thrombin. The increased conductance is likely due to PPACK bridging of surrounding residues into a compact, tetrahedral configuration (Figure S15, Supporting Information).^[^ ^52^ ^]^ DFT calculations also reveal a compact spatial distribution of frontier molecular orbitals, which predominantly contribute to charge transport through this region (Figure 3g, right part, see details in Experimental Section).

We also measured thrombin‐PPACK complexes randomly adsorbed on gold without DNA assistance (Figure S14b, Supporting Information). The average conductance was 10^−5.72^ G_0_ (σ = 0.33, R^2^ = 0.927; 144 curves, Figure 3e), again higher than that of the randomly deposited PPACK‐free thrombin, consistent with the results from the DNA‐origami‐confined protein/inhibitor complexes. However, the conductance distribution was notably broadened here due to the lack of a DNA template. Dual‐sample *t*‐tests yielded *t*‐values of −15.16 (bivalently anchored, *P* = 0.00265) and −4.13 (freely absorbed, *P* = 0.08713), confirming the significantly improved statistical confidence toward better‐resolved conformational changes in proteins based on the thrombin‐origami system. Taken together, these results clearly demonstrate that our strategy enables sensitive electrical readout of subtle structural transitions at the single‐molecule level.

### 2.6 Orientation Control for Single‐Molecule Electronic Measurement of Streptavidin

**Figure 4:**
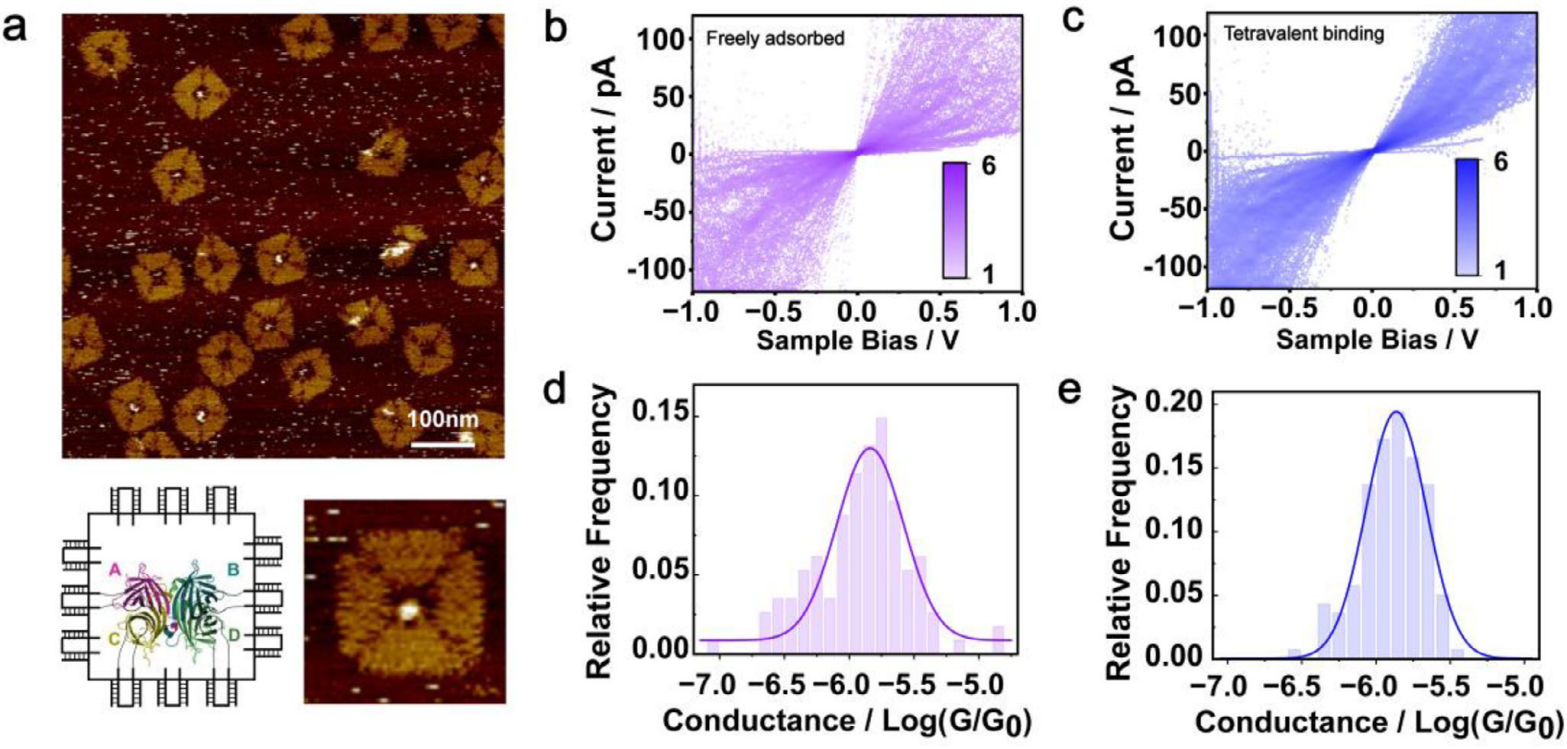
DNA‐origami‐based orientation strategy refines streptavidin (SA) alignment for single‐molecule measurements. a) Schematic and AFM images showing a DNA origami structure with four SA aptamers binding to the four biotin‐binding sites of a streptavidin tetramer. b) Intensity map of *I–V* curves from SA freely adsorbed on the Au surface, without aptamer anchoring. c) Intensity map of *I–V* curves from SA tetravalently anchored by four DNA origami‐linked aptamers. d) Conductance distribution of randomly adsorbed SA molecules, showing a relatively narrow peak. e) Conductance distribution of tetravalently anchored SA, with further reduced variance and a better‐fitted Gaussian shape, indicating improved orientation control by DNA origami. The color bar represents the number of counts (density) in each grid. Both voltage (x) and current (y) axes are divided into 151 grids.

To evaluate the general applicability of our strategy, we chose streptavidin (SA), a tetrameric protein structurally distinct from thrombin, to perform single‐molecule electric measurements. Unlike thrombin, which has a compact quasi‐globular shape, SA adopts a more anisotropic and symmetric architecture, often described as “disk‐like” due to its four subunits arranged in a D_2_‐symmetric configuration.^[^ ^53^ ^]^ This oblate‐shaped SA molecule may promote a more consistent adsorption geometry on Au surfaces, even in the absence of an external orientation control by DNA origami.^[^ ^54^ ^]^ To target SA, we replaced the thrombin‐binding aptamer pair with four identical nonhomologous random recombination (NRR)‐evolved SA aptamers,^[^ ^55^ ^]^ which specifically recognize the biotin‐binding sites on the subunits of SA. AFM imaging confirmed that the four protruding DNA helix arms installed inside the square origami cavity—each ending with an SA aptamer—can simultaneously bind to the SA tetramer (**Figure** **4**a). We first measured randomly deposited SA molecules (Figure 4b,d) and extracted conductance values from 114 *I–V* curves (±0.4 V), resulting in an average conductance of 10^−5.84^ G_0_ (σ = 0.26, R^2^ = 0.882; Figure 4d). Compared to thrombin untethered by the DNA origami, SA showed a significantly narrower conductance distribution, suggesting that SA naturally adopts a preferred orientation at the electrode interface.

We further measured tetravalently anchored SA on the DNA origami (Figure 4c,e) by C‐AFM. The resulting average conductance was 10^−5.86^ G_0_ (σ = 0.20, R^2^ = 0.973, 138 curves), comparable to that of the unconfined SA. Such a similarity indicates that tetravalent anchoring does not significantly alter the inherent orientation of SA on the Au surface. However, the reduced variance demonstrates that the origami‐based approach can further refine the protein orientation and improve the precision of single‐molecule electronic measurements, even for proteins that already favor a special adsorption geometry.

### 2.7 Precise Electronic Readout of Target Proteins in a Binary Protein Mixture

Aptamers have the ability to bind with molecular objects with high affinity and specificity, enabling the recruitment of desired proteins from a heterogeneous environment. We therefore assessed the capability of our DNA‐origami‐based platform in selectively isolating and electronically measuring special targets from a binary protein mixture.

**Figure 5:**
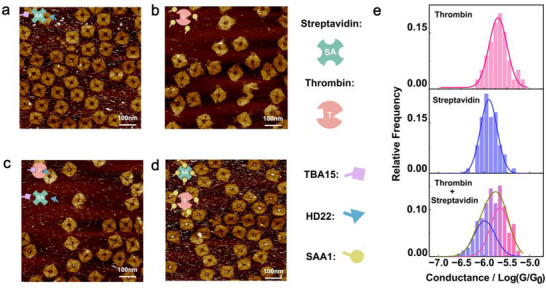
Selective recruitment and conductance measurement of target proteins from binary mixtures. a,b) AFM images showing negligible nonspecific protein binding when thrombin‐binding origami is incubated with streptavidin (SA) (a), or SA‐binding origami is incubated with thrombin (b). c,d), AFM images showing selective protein recruitment when thrombin‐binding origami (c) or SA‐binding origami (d) is incubated with a 1:1 thrombin‐SA mixture. Empty vs filled cavity ratios calculated from large‐area AFM images (Figure S16, Supporting Information): (a) 64:2, (b) 25:0, (c)17:12, (d) 24:37. Only origamis with intact structure are counted. e) Conductance histograms of individual protein junctions formed under mixed‐protein conditions. Top: thrombin‐binding origami; middle: SA‐binding origami; bottom: a mixture of thrombin‐binding and SA‐binding origami. The blue, pink, and brown curves are Gaussian fit of the data.

As a specificity control, we first incubated thrombin‐binding origami (5 nM) with streptavidin (SA, 100 nM), and SA‐binding origami (5 nM) with thrombin (100 nM). In both cases, AFM imaging revealed no detectable protein occupancy in the origami's central cavity (Figure 5a,b; Figure S16a,b, Supporting Information), indicating negligible nonspecific binding to non‐cognate aptamers.

We next incubated a 1:1 mixture of thrombin and SA (100 nM each) with either thrombin‐ or SA‐binding origami (5 nM). AFM images showed protein occupancy inside a substantial fraction of the DNA nanocavities (Figure 5c,d; Figure S16c,d, Supporting Information), evidencing a selective recruitment of corresponding proteins from the mixture. Single‐molecule conductance measurements of the isolated proteins (Figure S17a,b, Supporting Information) revealed G values of 10^−5.69^ G_0_ (σ = 0.20, R^2^ = 0.966, 80 curves) for thrombin (Figure 5e top) and 10^−5.90^ G_0_ (σ = 0.18, R^2^ = 0.953, 72 curves) for SA (Figure 5e middle). These results closely resemble those obtained from unmixed proteins (Figures 2h and 4e), signifying an excellent selectivity of the origami‐guided single‐molecule electric measurements.

To illustrate the potential for simultaneous recognition of multiple proteins, we mixed the thrombin‐ and SA‐binding origami structures before adding them to the binary protein solution (Figure S17c, Supporting Information). Double‐peak fitting of the resulting conductance histogram (Figure 5e bottom, 158 curves) resolved two distinct peaks at 10^−5.62^ G_0_ (σ = 0.21) and 10^−5.94^ G_0_ (σ = 0.25) with a high goodness of fit (R^2^ = 0.949), indicating a coexistence of both protein species and a successful discrimination of them based on molecular conductivities. By contrast, when thrombin and SA were randomly adhered to the Au surface without DNA origami guidance, the resulting conductance histogram (Figure S18, Supporting Information) showed poorly resolved peaks at 10^−5.70^ G_0_ (σ = 0.49) and 10^−5.83^ G_0_ (σ = 1.25) with significantly reduced fitting quality (R^2^ = 0.727). The seriously overlapped, broadened conductance peaks thus hinder a reliable recognition of the two proteins.

## 3 Discussion

We successfully developed a DNA‐origami‐guiding strategy for orientation‐controlled single‐molecule conductance measurements of proteins. This approach improves both the precision and the statistical reliability of electronic measurements and enhances the ability to distinguish subtle structural changes in proteins. We further demonstrated the applicability of the method across structurally distinct proteins, confirming its versatility.

Compared with previously reported protein conductance measurements using conductive atomic force microscopy (C‐AFM),^[^ ^56^, ^57^ ^]^ our approach ensures spatially dispersed, unambiguous single‐molecule detections. In contrast to other single‐molecule electronic techniques such as break junction^[^ ^9^ ^]^ or blinking‐based methods,^[^ ^10^ ^]^ our strategy does not require binding unit engineering or chemical functionalization of proteins to enable electrode attachment. This avoids potential disruption to protein structures and activities caused by site‐specific mutations.

It is worth noting that, ideally, such measurements should be conducted under aqueous buffers in order to capture dynamic conformational changes of proteins. However, the stringent requirement for probe insulation (<1 pA leakage current) currently exceeds the capabilities of available tip‐fabrication techniques, which can only achieve ≈10 pA leakage at best (for the C‐AFM probe). As such, this study focused on electric measurements in ambient air with 30% relative humidity. Under this condition, fluorescence spectra (Figure S19, Supporting Information) of dry protein films did not show significantly blue or redshifted emissions from the Trp residues compared to wet proteins, indicating marginal changes to the local electrostatic environments and the degree of solvent exposure for the Trp fluorophores.^[^ ^58^, ^59^ ^]^ Additionally, control experiments (Figure S20, Supporting Information) carried out in a nitrogen glovebox (0% humidity), where thrombin is expected to lose its structural water, showed a decreased average conductance of 10^−5.75^ G_0_ (σ = 0.175, R^2^ = 0.924, 62 curves). This result provides indirect evidence for the hydrated state of thrombin under actual measurement conditions with 30% humidity. These findings demonstrate that although the measurements were carried out in air, the proteins retained their conformations comparable to physiologically relevant states. In future work, we aim to further improve tip insulation technology to enable real‐time measurements of protein dynamics in aqueous buffers.

Furthermore, two main factors may contribute to uncertainty in contact position/angle during matrix‐patterned electronic measurements of the origami‐protein complexes. First, the conductive probe used has a tip radius of curvature of ≈25 nm, which is substantially larger than the thrombin molecule (≈3.5 nm in diameter) and comparable to the DNA origami's central cavity (≈20 nm; Figure S21, Supporting Information), limiting the spatial precision of the setup. Second, positional drift happens after the AFM imaging, which may alter the contact location during a subsequent electrical measurement. Reducing the imaging area, increasing the spatial resolution of the scanning matrix, or using sharper probes could help mitigate these issues and improve measurement accuracy.

To improve measurement reproducibility, we used Pt‐Ir‐coated probes, a wear‐resistant material suitable for repeated scans. Kelvin probe force microscopy (KPFM) calibration of the Pt–Ir coated probe and an Au coated probe against the same HOPG reference (Figure S22, Supporting Information) revealed an average surface potential of 0.117 V for Pt‐Ir and 0.237 V for Au. The small work function difference (0.12 eV) between the two probes, combined with the symmetric features of the measured *I–V* curves, suggests that the asymmetry in electrode materials has a negligible effect on the conductance measurements.

## 4 Conclusion 

We have established a DNA‐origami‐based strategy that promotes orientation‐controlled protein–electrode coupling, enabling single‐molecule electronic readout with improved reproducibility and reliability. Benefiting from the precisely aligned DNA aptamers inside a square‐shaped DNA origami nanocavity for protein capturing, stable and oriented attachment of individual protein molecules between two electrodes has been achieved, allowing for precise electrical measurements on single proteins. The use of an aptamer‐decorated nanocavity for protein encapsulation also avoids blocking the current pathway during a measurement. In particular, bivalent aptamer binding biases thrombin toward more uniform and stable orientations, which significantly narrows its conductance distribution and thus improves signal reproducibility. The confined protein/electrode coupling geometry also allows a detection of subtle conformational changes in thrombin induced by Na^+^ binding or PPACK inhibition, reflected by significant conductance shifts. Further application of this method to streptavidin (a structurally distinct tetramer protein), based on tetravalent aptamer anchoring, verifies the generality of the method. Moreover, the DNA‐based platform supports selective recruitment and electrical readout of two target proteins directly from their binary mixtures, with resolvable conductance signals from each species. This capability suggests the potential of electronically probing multiple protein species at the single‐molecule level, pointing toward future single‐entity electrical measurements in more complex and realistic protein environments. Our work establishes a versatile framework for high‐precision, label‐free electronic characterization of individual proteins. Future work toward expanded aptamer‐protein combinations and real‐time electrical readout could further its application in bioelectronics and molecular diagnostics.

## Conflict of Interest

The authors declare no conflict of interest.

## Author Contributions

Z.J., Z.D., and Y.L. designed the research. Z.J. performed the experiments and conducted theoretical calculations. Z.J., Y.L., Z.D., and J.L. performed the data analysis and wrote the paper. All authors discussed the results and commented on the manuscript.

## Supporting information



Supporting Information

## Data Availability

The data that support the findings of this study are available from the corresponding author upon reasonable request.
