# Supplementary material for: DNA‐Guided Robust Single‐Protein Electronic Readout
Source: Adv Sci (Weinh). 2025 Dec 17;13(12):e16711. doi: 10.1002/advs.202516711 (PMC12948236; doi:10.1002/advs.202516711)
Supplement: Supplementary file 1 — Supporting Information [file ADVS-13-e16711-s001.docx]

**Supplementary Information**

**DNA-Guided Robust Single-Protein Electronic Readout**

Zi-Yi Ju, ^a b^ Zhao-Xiang Deng ^a b*^, Yue-Qi Li^a*^ and Jing-Hong Li ^a b c*^

^a^ Hefei National Research Center for Physical Sciences at the Microscale, University of Science and Technology of China, Hefei 230026, China

^b^ Department of Chemistry, School of Chemistry and Materials Science, University of Science and Technology of China, Hefei 230026, China

^c^ Department of Chemistry, Key Lab of Bioorganic Phosphorus Chemistry and Chemical Biology, Tsinghua University, Beijing 100084, China

*corresponding author: zhxdeng@ustc.edu.cn

yueqili@ustc.edu.cn

jhli@mail.tsinghua.edu.cn

**Table of Contents**

**Methods2**

Supplementary Figures6

**DNA origami sequence** **16**

Supplementary References27

**Methods**

***Materials***

Magnesium acetate (MgAc_2_, 99.5%) were purchased from HWRK CHME, Inc. Potassium chloride (KCl, 99.99%) were purchased from Shanghai Macklin Biochemical Technology Co., Ltd (Macklin, China). Lithium chloride (LiCl, 99.9%), Sodium chloride (NaCl, 99.5%), Dimethyl sulfoxide (DMSO, > 99%), Gold (Au, 99.999%) were purchased from Shanghai Aladdin Biochemical Technology Co., Ltd. 50 × TAE buffer (2 mol/L Tris -acetic acid, 50 mmol/L EDTA, pH 8.0) and 1 × TE buffer (low EDTA, pH 8.0) were purchased from Sangon Bioengineering Technology Service Co., LTD. Ultrafiltration centrifuge tubes (UFC5100BK-1,0.5ml, 100KD) were purchased from Merck Millipore. Human alpha-Thrombin Native Protein (RP-43100, 6.8mg/mL) were purchased from Invitrogen. Streptavidin from streptomyces avidinii (SA, ≥13 units/mg protein) were purchased from Sigma-Aldrich Co. LLC. D-Phe-Pro-Arg-chloromethylketone (PPACK, > 98.00%) were purchased from Glpbio. β-Ala-Gly-Arg-pNA (99.10%) were purchased from MedChemExpress Co., Ltd. M13mp18 Single-stranded DNA (250 µg/ml) were purchased from New England Biolabs, Inc. All the reagents were used as received without further purification.

Mica sheets (Grade V 1) were purchased from TED PELLA, INC. AFM probe MULTI75GB (Gold Overall Coating) were purchased from Innovative Solutions Bulgaria Ltd, and other probes were purchased from Bruker Corporation.

***DNA Origami Folding***

DNA staple strands were custom-synthesized by Sangon Biotech Co., Ltd. (Shanghai, China), and purified by HAP (for unmodified strands) or HPLC (for thiol-modified strands). The M13mp18 single-stranded DNA scaffold was obtained from New England Biolabs. Each DNA origami folding solution contained 10 nM scaffold, 100 nM unmodified staple strands, 125 nM thiol-modified edge staples, 100 nM unmodified cavity staples, 500 nM cavity staples for aptamer conjugation, and 1 µM of each DNA aptamer. A folding buffer containing 40 mM Tris- acetic acid, 1 mM EDTA, 12.5 mM MgCl_2_, and 5 mM KCl (pH 8.0) was employed. For experiments involving different ionic environments, 140 mM NaCl or LiCl was added. Folding was carried out using a thermal ramp protocol: initial incubation at 70 °C for 5 min, followed by a temperature decrease from 65 °C to 50 °C at a rate of 0.1°C every 12 s, and then from 50 °C to 20 °C at a rate of 0.1 °C every 6 min. To prevent product aggregation, thiolated DNA strands retained their C_3_-SH protecting groups during folding. The resulting DNA origami structures were able to lie flat on surfaces with -S-S- functionalized edges for their anchoring to gold substrates.

***DNA Origami Purification***

Folded DNA origami structures were purified at 4 °C using Amicon Ultra 0.5 mL centrifugal filters with a 100 kDa molecular weight cutoff (UFC5100BK, Millipore). Prior to use, the filter membrane was preconditioned by centrifuging 400 µL of the folding buffer at 2500×g for 3 min. Subsequently, 50 µL of the folded DNA origami was mixed with 450 µL of the folding buffer, loaded into the filter, and centrifuged at 2500×g for 12 min. The purified sample was volumed to 500 µL by adding folding buffer and the centrifuged again. The step was repeated twice. The purified sample was then recovered by inverting the filter into a clean 0.5 mL tube and centrifuging at 1000×g for 3 min. The final sample was stored at 4°C before use.

***Protein Binding to DNA Origami***

Human α-thrombin and streptavidin from streptomyces avidinii were diluted to 1.85 µM and 900 nM, respectively, in the folding buffer. For streptavidin binding, 5.5 nM DNA origami was incubated with 72 nM streptavidin at 37 °C for 2 h. For thrombin binding, 10 nM DNA origami was incubated with 74 nM thrombin under the same conditions. To achieve PPACK binding to thrombin, PPACK was dissolved in DMSO, diluted by the folding buffer, and mixed with thrombin at a 1:1.1 molar ratio. The mixture was incubated at 37°C for 1 h, followed by addition to the DNA origami sample to form protein/origami complexes.

***Preparation of Atomically Flat Gold Substrates***

For C-AFM measurements, a 160-nm-thick gold film was deposited onto freshly cleaved mica (TED PELLA) using electron-beam evaporation (MB-Unlvap), followed by annealing at 300 °C for 12 h. A drop of UV-curable adhesive was applied between the gold film and a glass slide, followed by curing under 365 nm UV light for 3 min. The gold film was peeled off from the mica along with the glass slide. Conductive gel was applied along the edges of the gold film to ensure a low-resistance connection with the metal stage.

***Sample Preparation for Conductive Atomic Force Microscopy Measurements***

To deposit protein-free and thrombin-loaded DNA origami structures onto a gold (Au) surface, a 10 µL sample was applied to the gold substrate, incubated for 1 min, washed with 200 µL of deionized water, and dried under a gentle air stream.

To prepare a gold surface adsorbed with free proteins (without DNA origami confinement), the gold surface was first incubated with the protein for 2 minutes and then extensively rinsed with deionized water, leaving partial exposure of the Au surface.

***Atomic Force Microscopy Imaging and Conductance Measurements***

For AFM imaging in liquid, a 3 µL sample solution was deposited onto freshly cleaved mica. After an adsorption for 30 s, 60 µL of a folding buffer was added to cover the mica surface. Imaging was immediately performed in liquid using a MultiMode8 AFM (Bruker) with a ScanAsyst-Fluid+ probe. For AFM imaging in air to confirm intact origami structures on an Au substrate, a ScanAsyst-Air probe (Bruker) was used.

In the case of C-AFM measurements, I-V curves were obtained by a Tunneling AFM system (Pre-amplifier: 10 pA/V) using a SCM-PIC-V2 probe (Bruker). Ambient humidity was maintained at approximately 30%. In contact mode, origami structures on the gold electrode were located, and the AFM tip was brought into contact with a bias voltage of ±1 V. Several forces were tested, and the optimal force of 232 pN was used in all subsequent measurements. The force was calculated by F = Setpoint (0.2 V) × Spring constant (0.1 N/m) × Deflection sensitivity (11.6 nm/V). To reduce tip drift and positioning error, I-V curves were collected over a 5×5 lattice centered on the origami. Only when the points surrounding the center of the DNA origami exhibited weak I-V responses (maximum current < 50 pA), and the central point displayed a markedly higher I-V response (distinct from the large signals typically observed on bare Au surfaces), was the central point included in the statistical analysis of single-molecule thrombin conductance. Conductance was extracted by linear fitting of the I-V curves in the range of -0.4 V to +0.4 V using OriginPro 2023 (OriginLab Corporation). AFM images were analyzed by a NanoScope Analysis software (Bruker).

***Fluorescence spectroscopy characterization***

To a solution containing 18.5 µM thrombin dispersed in a folding buffer, was added 140 mM NaCl or LiCl, following by an incubation at 37 °C for 1 h. Fluorescence spectra ranging from 300 nm to 450 nm (step size: 1 nm) were recorded at 37 °C under an excitation wavelength of 280 nm.

***Enzyme activity assay***

The following reaction was involved to quantitate the enzyme activity of thrombin:

Thrombin

β-Ala-Gly-Arg-pNA → β-Ala-Gly-Arg-OH + pNA

To prepare a substrate stock solution, 10 mg of β-Ala-Gly-Arg-pNA was dissolved in 500 µL of DMSO to reach a concentration of 23.67 mM. Thrombin was dissolved in the folding buffer to make a 1 µM enzyme stock solution.

The total reaction volume was 150 µL, each prepared as follows:

Control group: Blank control: 5 µL substrate stock + 145 µL folding buffer; Positive control: 5 µL substrate stock + 15 µL thrombin stock (1 µM) + 130 µL folding buffer.

Experimental group: 30 µL thrombin stock was mixed with 10 µL of a 3.3 µM PPACK solution, followed by an incubation at 37 °C for 1 h; 5 µL substrate stock was then combined with 20 µL of the pre-incubated protein/inhibitor mixture and a 125 µL folding buffer.

UV absorbance of all samples at 405 nm were immediately recorded at 37 °C for a total duration of 2 h (10 s interval).

***Computational methods***

Density functional theory (DFT) calculations of the electrostatic potential distribution and electronic structure were performed using the Dmol^3^ module of Materials Studio (MS). The exchange-correlation interaction was treated by the generalized gradient approximation (GGA) with the Becke-Lee-Yang-Parr (BLYP) function. All protein structural fragments were derived from PDB, and the residues were saturated with H atoms. Spin unrestricted was chosen. DFT Semi-core Pseudopotential (DSPP) was applied for core treatment considering the relativistic effects. Considering the existence of hydrogen bonds in the system, the double numerical plus polarization (DNP) was set as basis for further calculation. The self-consistent (SCF) field tolerance was set to 1×10^−6^ Ha in this study.

**Supplementary Figures**


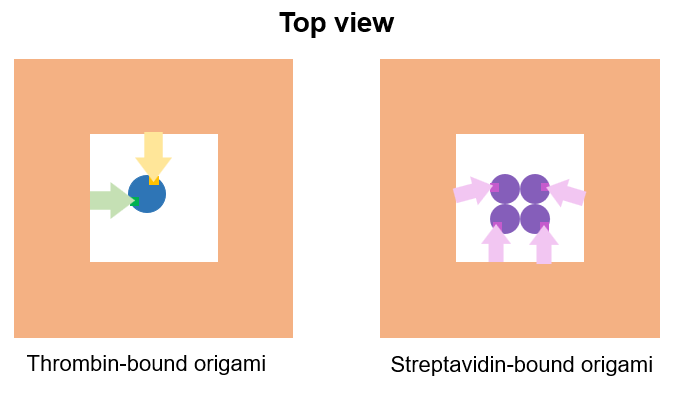


**Figure S1.** Top-view schematic of thrombin-bound (left) and streptavidin-bound (right) DNA origami. Orange squares represent DNA origami with a central cavity; arrows indicate aptamers extending inward; circles denote proteins; small squares indicate aptamer binding sites on the protein surface (based on PDB structure (5EW1) of thrombin bound to TBA15 and HD22, and the reported biotin-binding sites of streptavidin^[1]^).


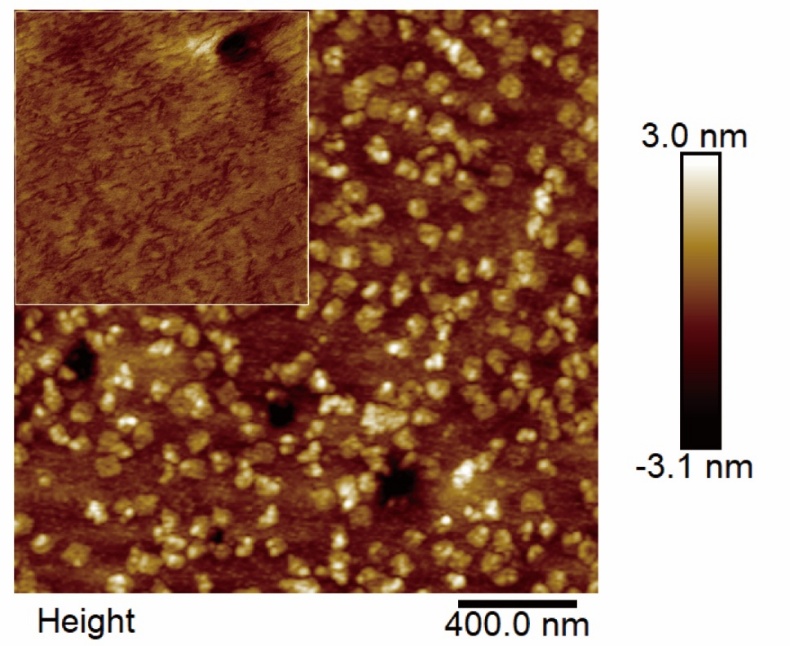


**Figure S2.** Contact-mode atomic force microscopy (AFM) imaging of the origami-modified Au surface and a bare Au surface (inset) with a C-AFM probe. A low force setpoint (232 pN) and a reduced number of sampling lines (256) were used to minimize tip-induced damage during contact-mode imaging. These settings, however, result in lower imaging resolution.


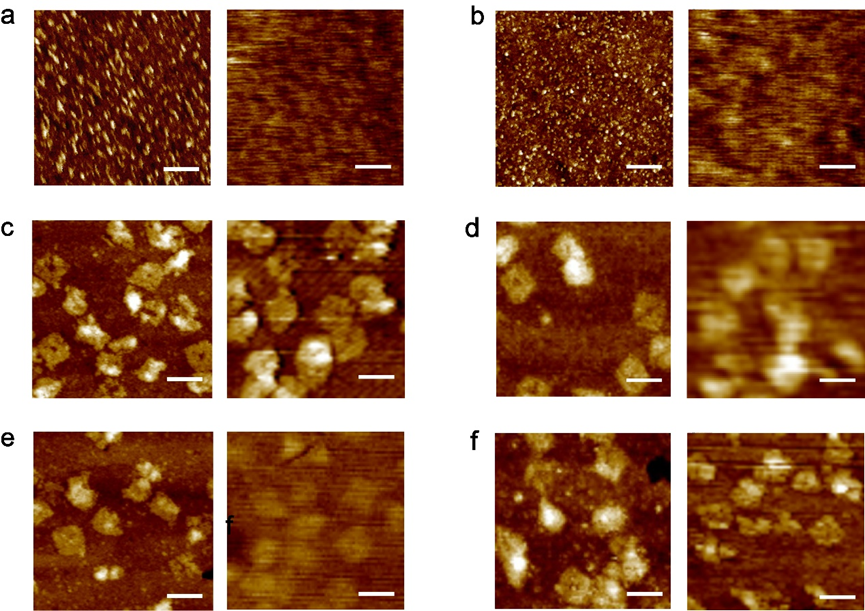


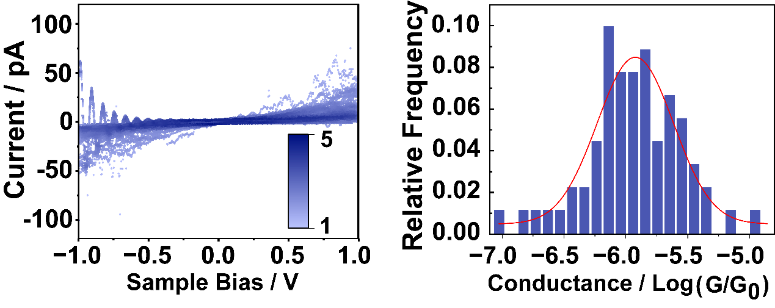
Figure S3. AFM topographic images obtained with a high-resolution probe(left) and with a C-AFM probe(right) at Au surface in the air of (a) freely adsorbed thrombin, (b) freely adsorbed streptavidin, (c) DNA origami with two thrombin aptamers, (d) DNA origami with four SA aptamers, (e) origami-thrombin complexes, (f) origami-streptavidin complexes. Scale bar:100nm.

**Figure S4.** Conductance measurement of DNA origami without protein binding. The intensity map of I-V curves (**left**, n = 66) recorded at 232 pN from DNA origami constructs without protein binding. The corresponding one-dimensional conductance histogram (**right**) was obtained by linear fitting within ±0.4 V. The average conductance is 10^-7.18^ G_0_, with σ = 0.31 and R^2^ = 0.866.


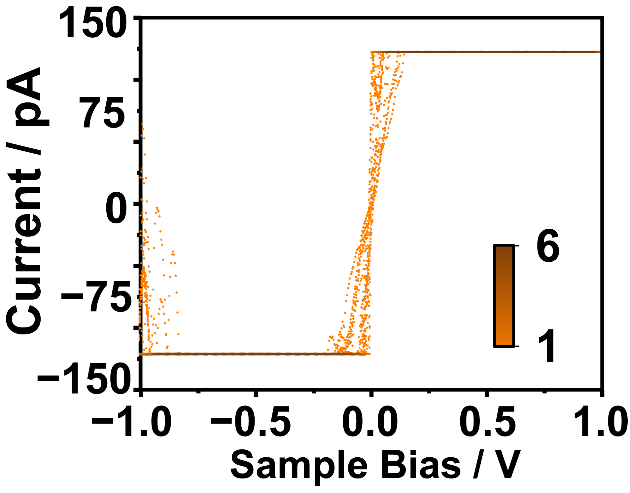


**Figure S5.** The intensity map of I-V curves recorded from origami carrying TBA15 and HD22 aptamers. n = 38.


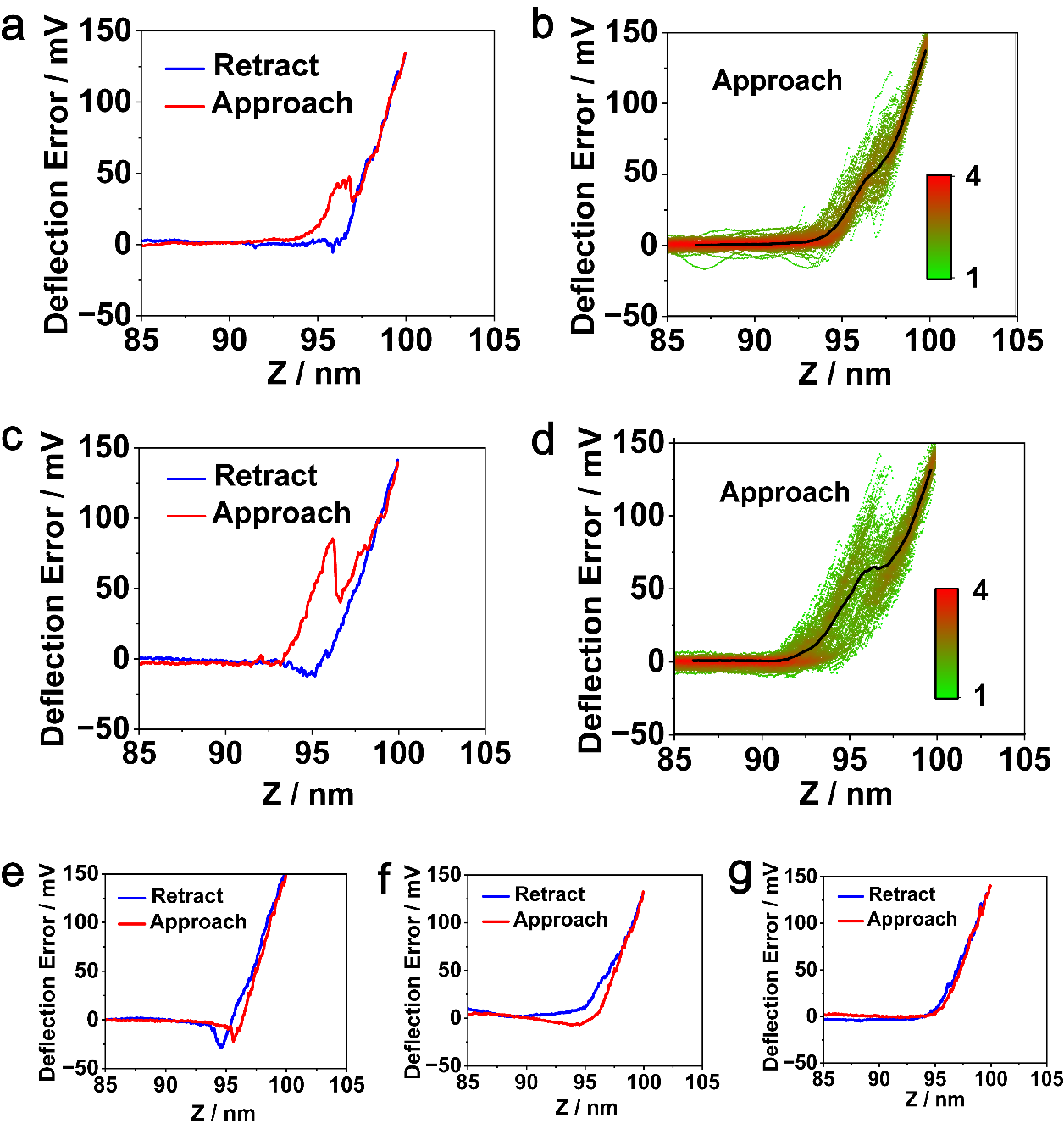


Figure S6. Force-indentation measurements with a high-resolution probe (2 nm radius of curvature): (a) thrombin (bivalent binding) and (b) intensity map of 92 curves showing an average threshold force of 343 pN (calculated as F = Average Deflection Error (42.25 mV) × Spring Constant (0.7 N/m) × Deflection Sensitivity (11.6 nm/V)), an average deformation of 2.46 nm (ΔZ_Average_ – ΔX_tip_ = 2.95 nm – 0.49 nm), and a remaining height of 1.04 nm (3.5 nm apparent height of thrombin – 2.46 nm deformation), the black line is the averaged curve; (c) streptavidin (tetravalent binding) and (d) intensity map of 88 curves showing an average threshold force of 468 pN (calculated as F = Average Deflection Error (57.65 mV) × Spring Constant (0.7 N/m) × Deflection Sensitivity (11.6 nm/V)), an average deformation of 2.33 nm (ΔZ_Average_ – ΔX_tip_ = 3.00 nm – 0.67 nm), and a remaining height of 1.67 nm (4.00 nm apparent height – 2.33 nm deformation), the black line is the averaged curve; (e) mica surface; (f) central cavity of TBA15/HD22 origami; (g) surface of TBA15/HD22 origami. The color bar represents the number of counts (density) in each grid. Both voltage (x) and current (y) axes are divided into 151 grids.


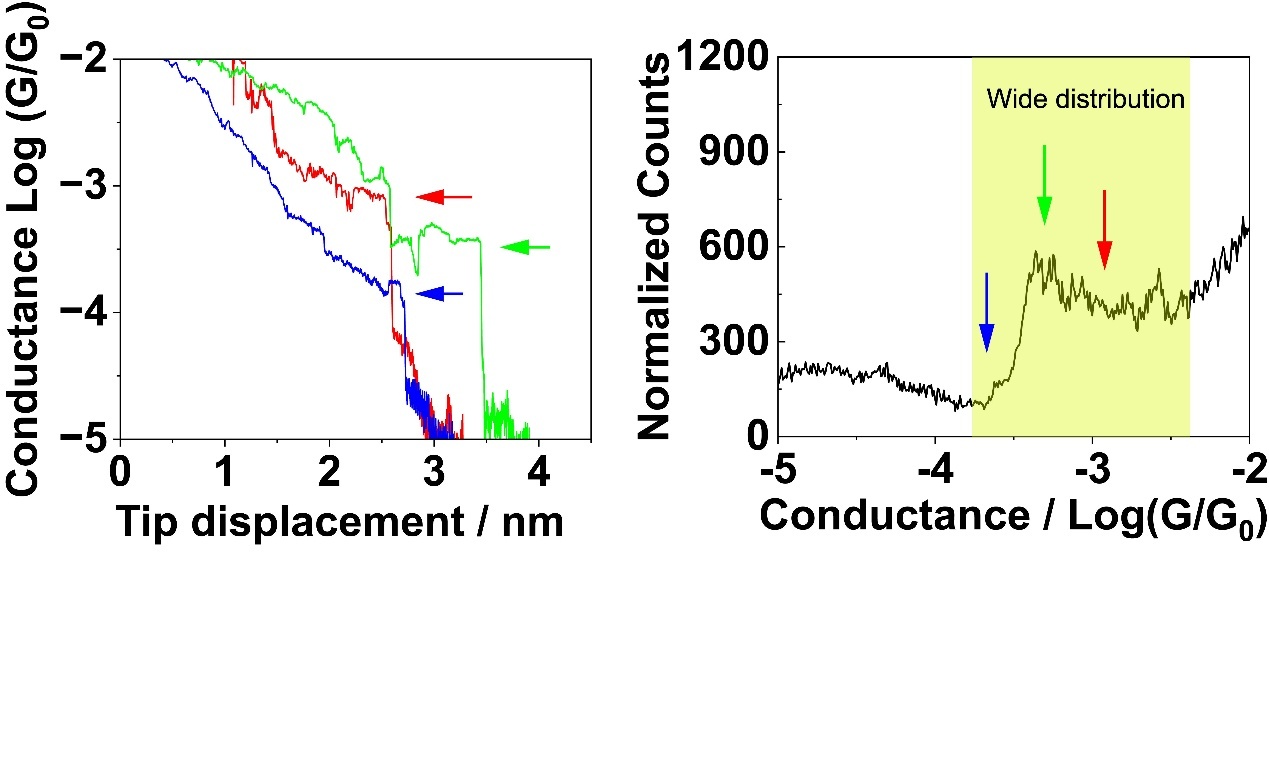


Figure. S7. Scanning tunneling microscopy break-junction measurements of single thrombin molecules. (a) Representative individual conductance-distance traces. (b) Conductance histograms of curves exhibiting plateaus.


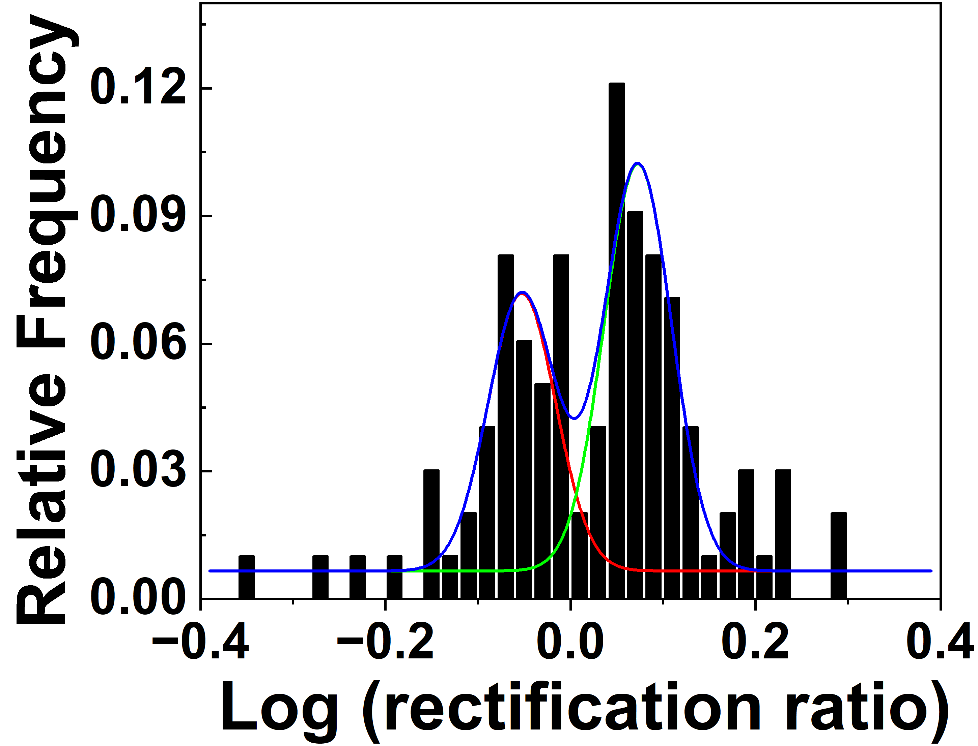


Figure S8. Histogram of log (rectification ratio, RR) for bivalently bound thrombin. The mean log (RR) values are -0.053 and 0.073, corresponding to RR values of 0.89 and 1.18. RR =$\left| \frac{I_{+0.4V}}{I_{-0.4V}} \right|$.


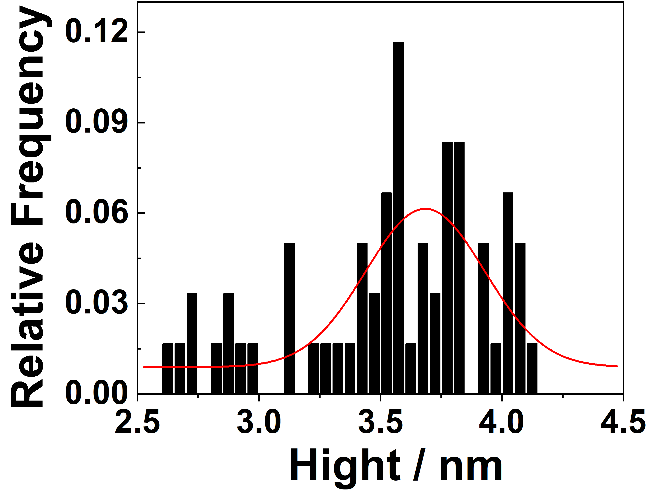

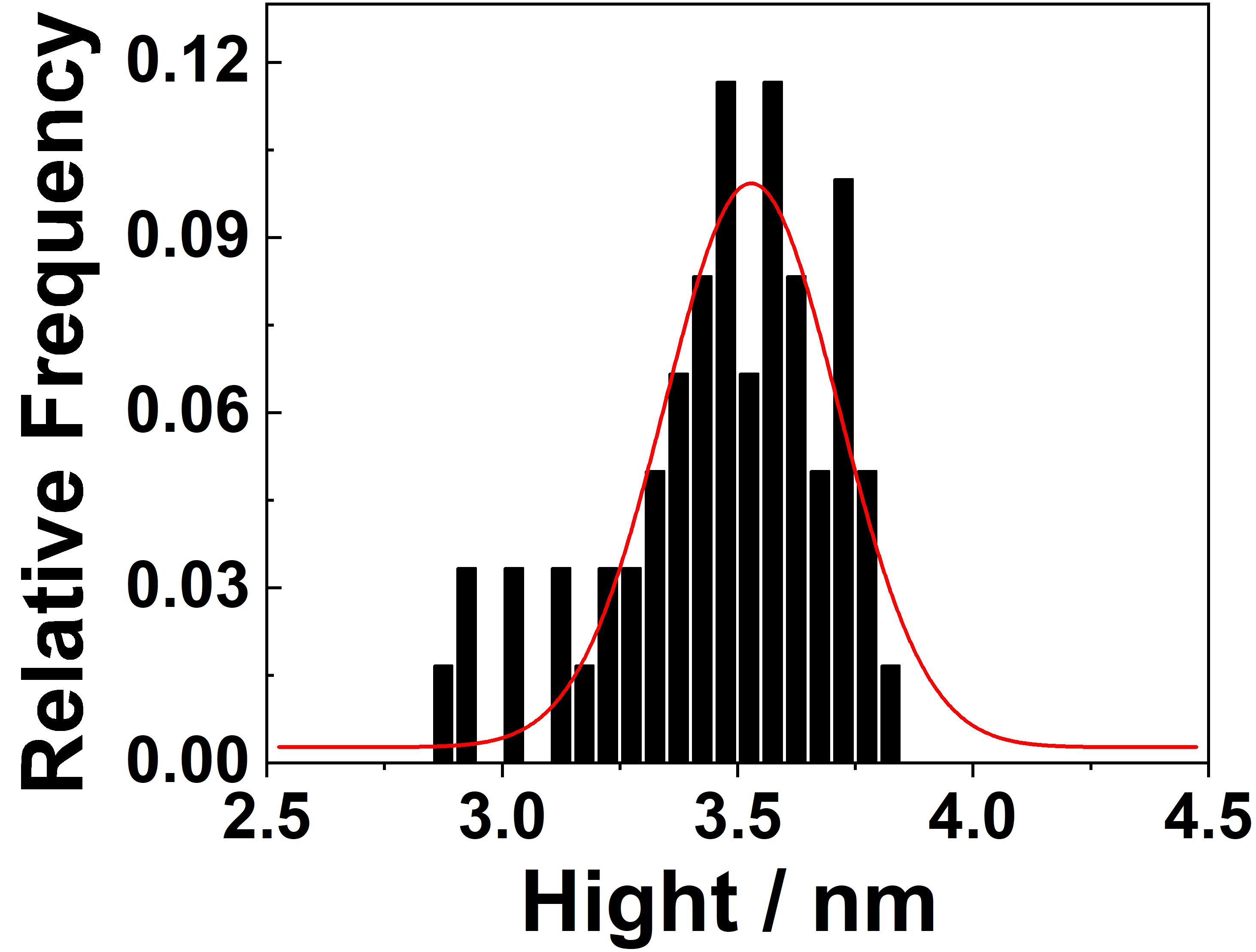


Figure S9. AFM height distributions of thrombin tethered bivalently (left, R^2^ = 0.84, σ = 0.18, n = 60) and monovalently (right, R^2^ = 0.43, σ = 0.24, n = 60) by DNA origami.


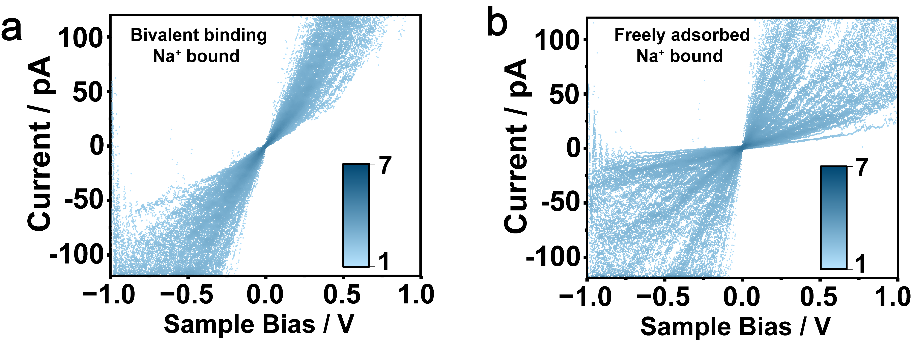


**Figure S10.** The intensity map of I-V curves recorded from thrombin with Na^+^ binding (Left: bivalent binding, n = 122. Right: Freely adsorbed, n = 136). The color bar represents the number of counts (density) in each grid. Both voltage (x) and current (y) axes are divided into 151 grids.


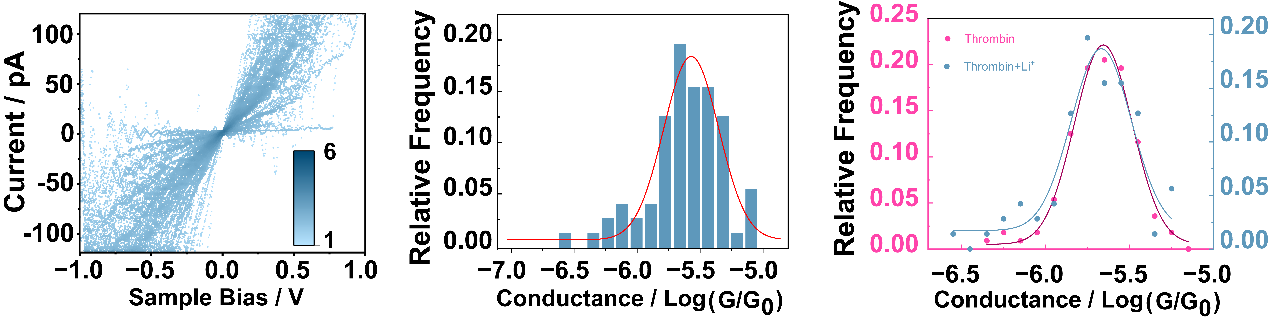


**Figure S11.** Single-molecule conductance of thrombin in the presence of Li^+^. The intensity map of I–V curves **(left)** recorded from thrombin bivalently anchored to DNA origami via two aptamers in the presence of 140 mM Li^+^. Corresponding one-dimensional conductance histograms **(middle)** were obtained by linear fitting within ±0.4 V. Average conductance :10^-5.67^ G_0_. Conductance histograms of bivalently anchored thrombin molecules (right) in the presence of Li^+^ **(blue)** shows no significant (T = -2.81, p = 0.16241) shift compared to thrombin in the presence of Na^+^ (10^-5.66^ G_0_, pink). The color bar represents the number of counts (density) in each grid. Both voltage (x) and current (y) axes are divided into 151 grids.


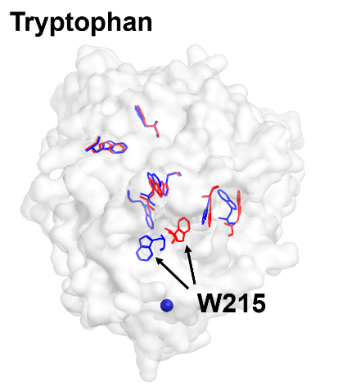

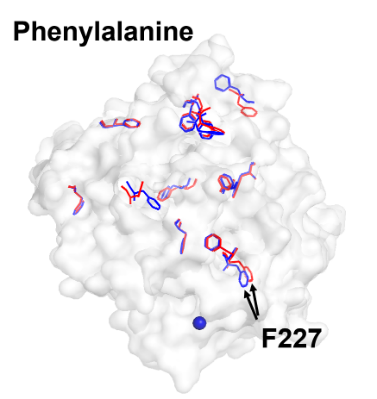

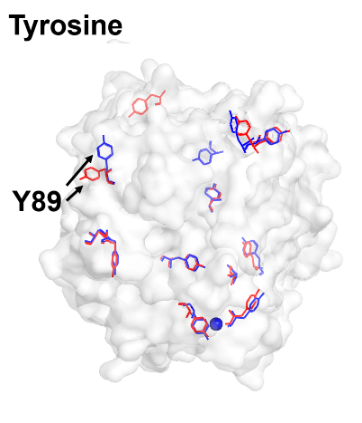


**Figure S12.** Na^+^-induced structural change of thrombin probed by modeling. Structural models illustrate the orientation of aromatic residues in the presence (blue, PDB:1SG8) and absence (red, PDB:1SGI) of Na^+^ ions (represented as blue spheres). The comparison highlights conformational changes induced by Na^+^ binding when thrombin is anchored via DNA origami on the gold surface. Structures drawn by Pymol from XRD structures.^[2]^


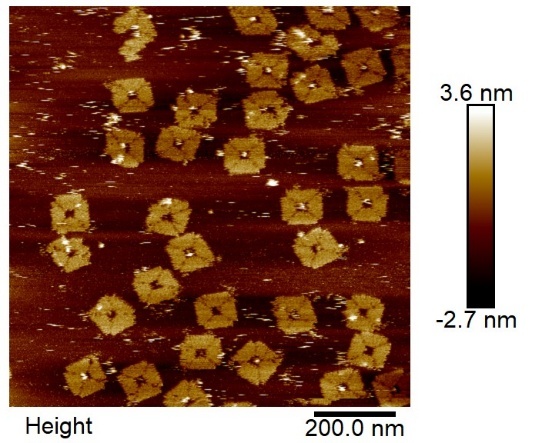
**
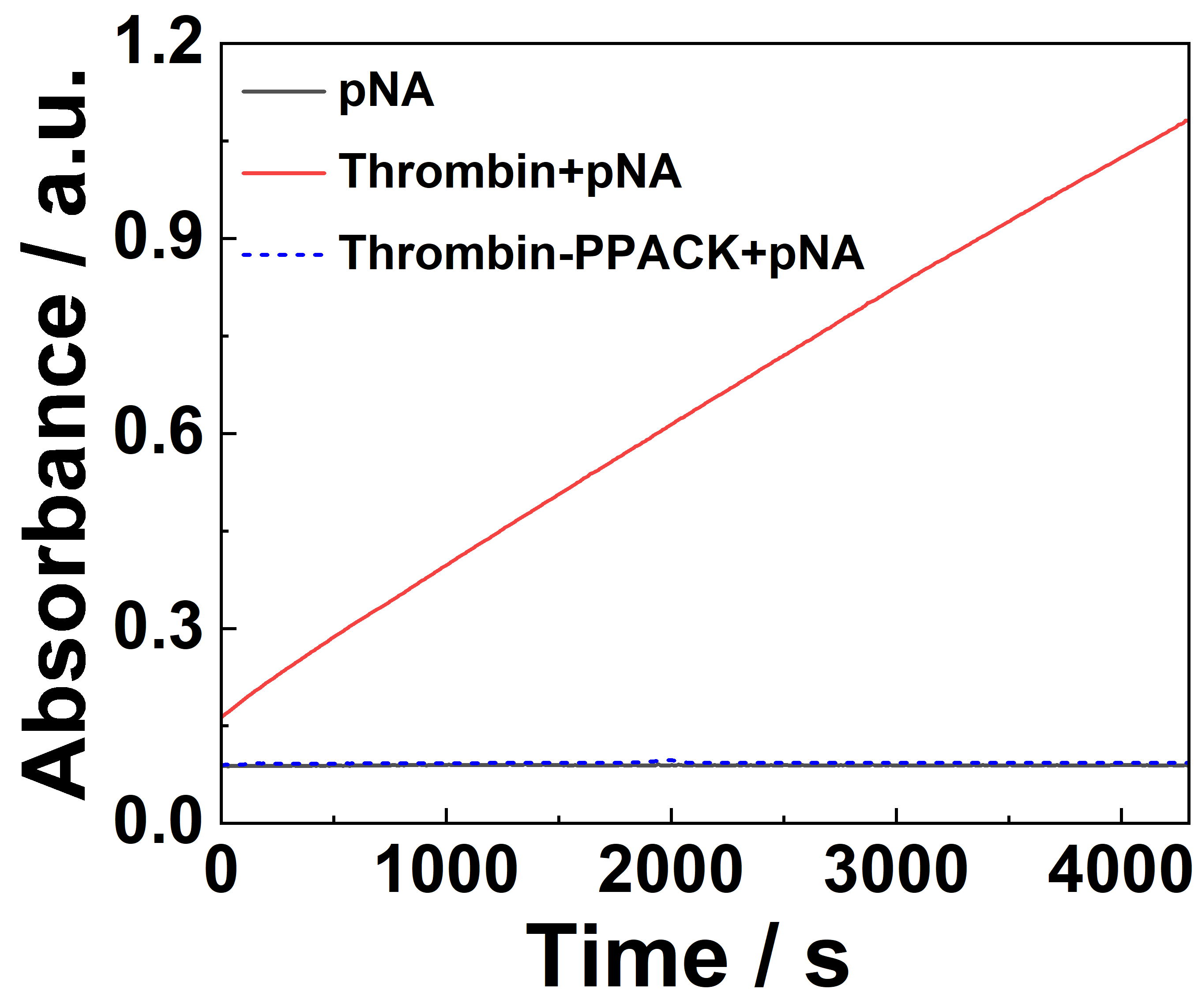
**

**Figure S13.** Successful capture of PPACK-inhibited thrombin was confirmed by AFM imaging and enzyme activity assay. **Left:** AFM image of DNA origami incubated with PPACK-inhibited thrombin on mica in imaging buffer. **Right:** enzyme activity assay demonstrates that PPACK blocks the active site of thrombin, preventing the binding of chromogenic substrate β-Ala-Gly-Arg-pNA and following cleavage of the UV-sensitive group pNA (Absorbance: 405nm).


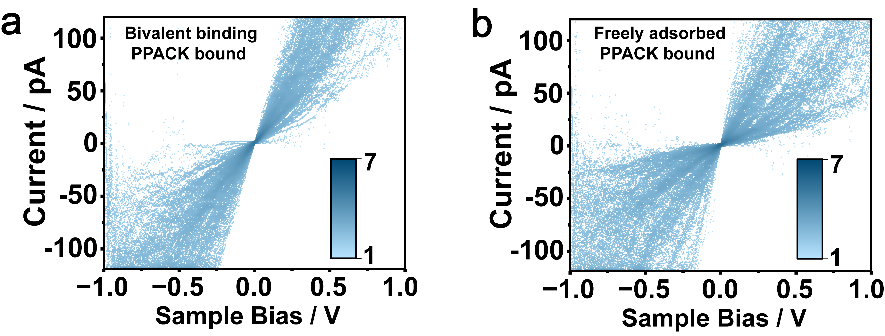


**Figure S14.** The intensity map of I-V curves recorded from thrombin with PPACK binding (**Left**: bivalent binding, n = 134. **Right**: Freely adsorbed, n = 144). The color bar represents the number of counts (density) in each grid. Both voltage (x) and current (y) axes are divided into 151 grids.


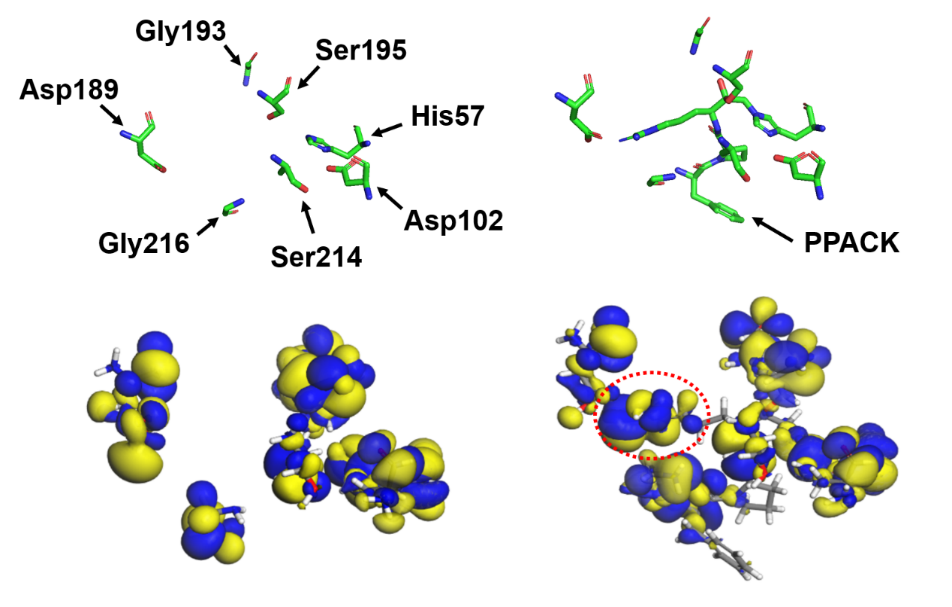


**Figure S15.** Calculations of the arrangement of residues in thrombin active site fragment (PDB: ISHH).^[3]^


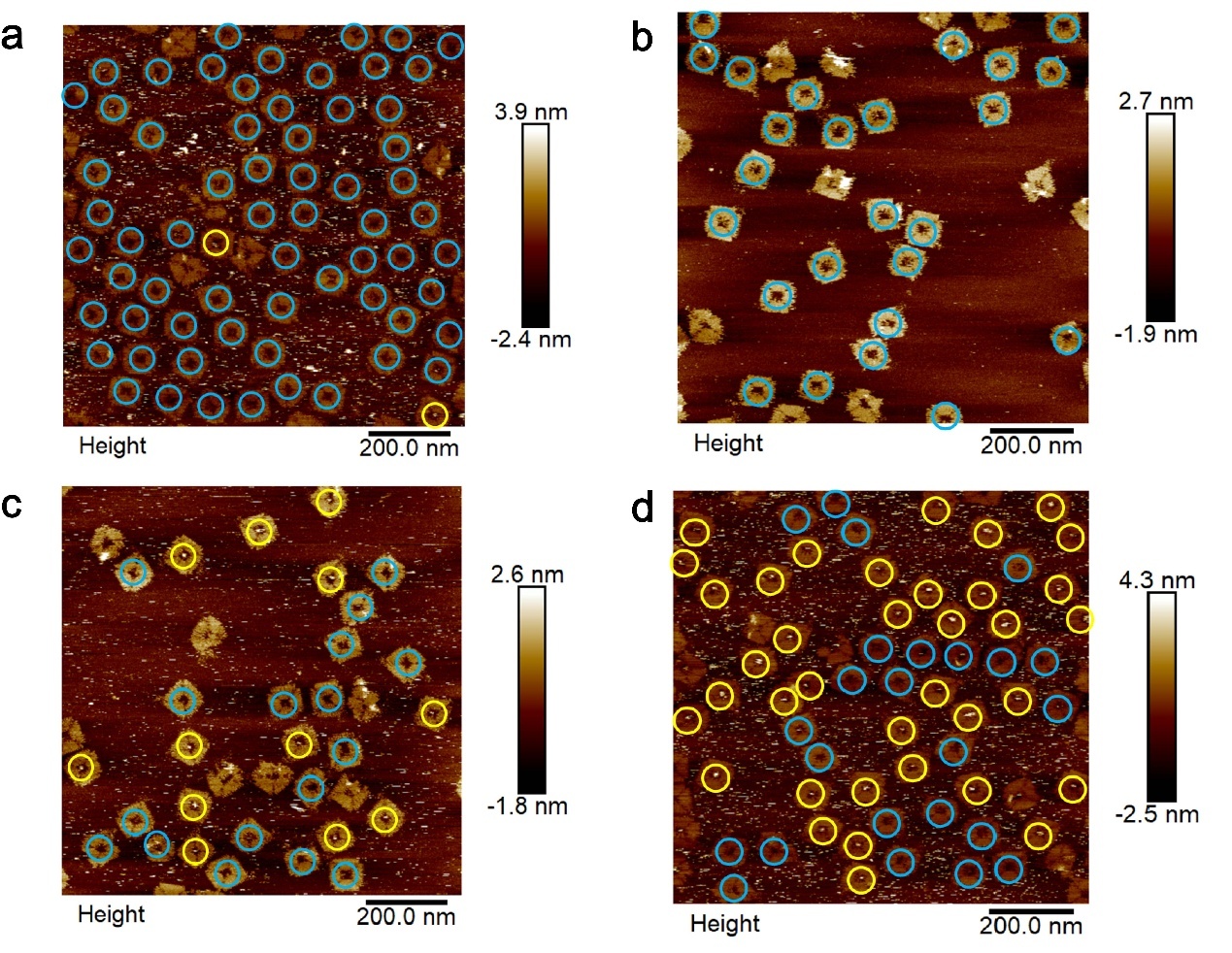


Figure S16. Large-area AFM images. (a, b) AFM images showing negligible nonspecific protein binding when thrombin-binding origami is incubated with streptavidin (SA) (a), or SA-binding origami is incubated with thrombin (b). (c, d) AFM images showing selective protein recruitment when thrombin-binding origami (c) or SA-binding origami (d) is incubated with a 1:1 thrombin-SA mixture. Ratios of empty to protein-filled cavities calculated from large-area AFM images: (a) 64:2, (b) 25:0, (c) 17:12, and (d) 24:37. Only origamis with intact structure are counted.


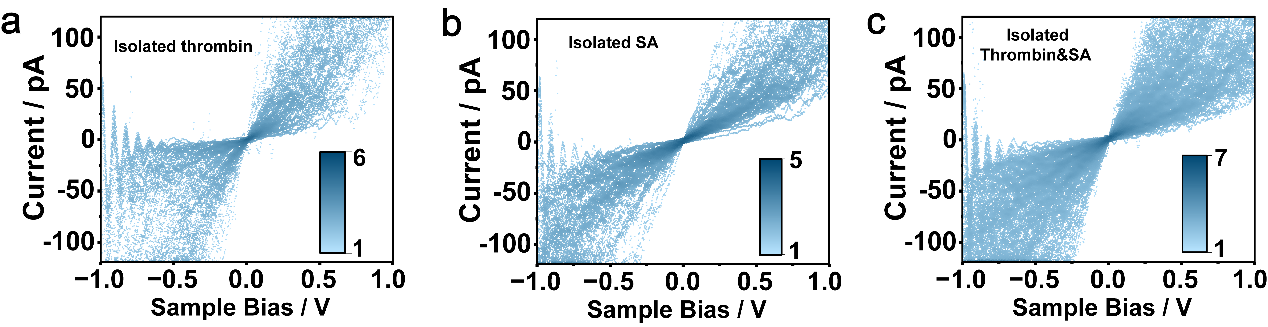


**Figure S17.** The intensity map of I-V curves recorded from (**a**): isolated thrombin (n = 80), (**b**) isolated SA (n = 72) and (**c**) isolated thrombin and SA (n = 158). The color bar represents the number of counts (density) in each grid. Both voltage (x) and current (y) axes are divided into 151 grids.

**
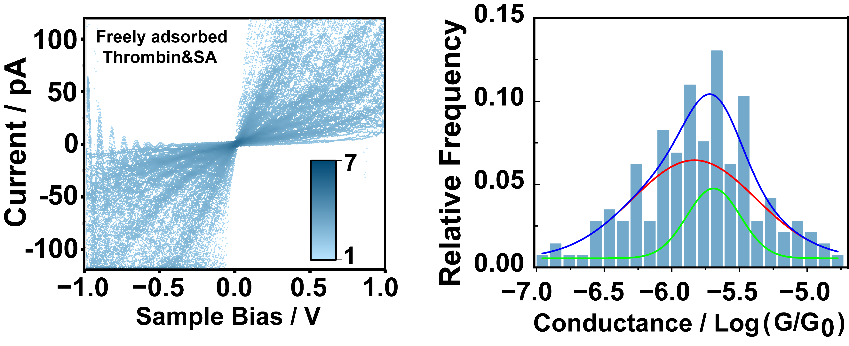
**

**Figure S18.** The intensity map of I-V curves **(left)** recorded from binary protein mixture (n = 146), and corresponding one-dimensional conductance histograms **(right)** obtained from linear fitting within ±0.4 V, showing two poorly resolved peaks at 10^-5.70^ G_0_ (green) and 10^-5.83^ G_0_ (red) (R² = 0.727). The color bar represents the number of counts (density) in each grid. Both voltage (x) and current (y) axes are divided into 151 grids.


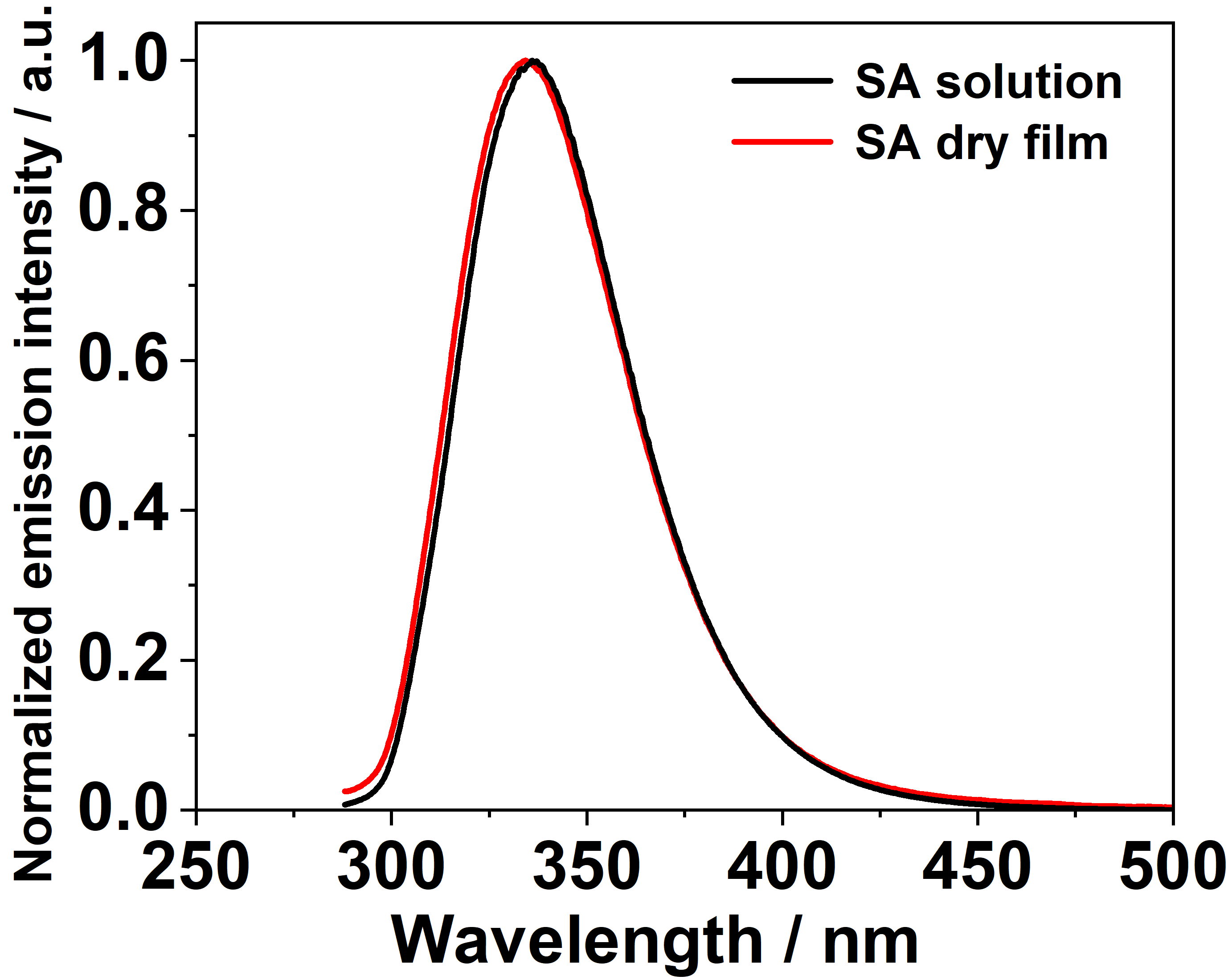

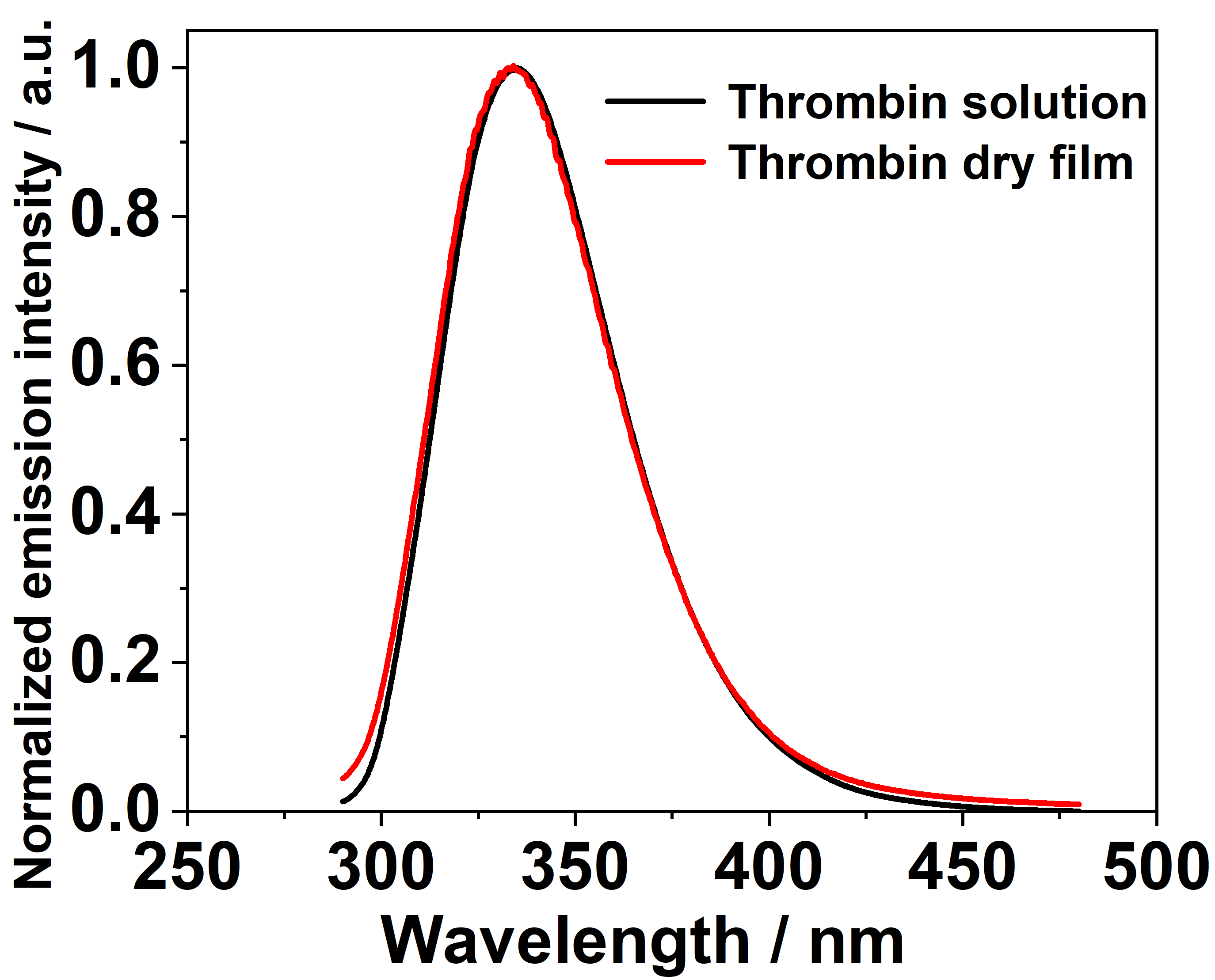


**Figure S19.** Normalized fluorescence emission spectra (measured using a Fluorescence Spectrophotometer F-7000, Hitachi) of thrombin **(left)** and SA **(right)** in solution (900 nM) and as a dry film on a gold substrate (prepared from a 30 μM solution and measured under ambient conditions with 30% relative humidity). Excitation wavelength: 275 nm. Emission maxima for thrombin appear at 335.0 nm (solution) and 334.0 nm (dry film), and for SA at 336.0 nm (solution) and 334.4 nm (dry film).

**
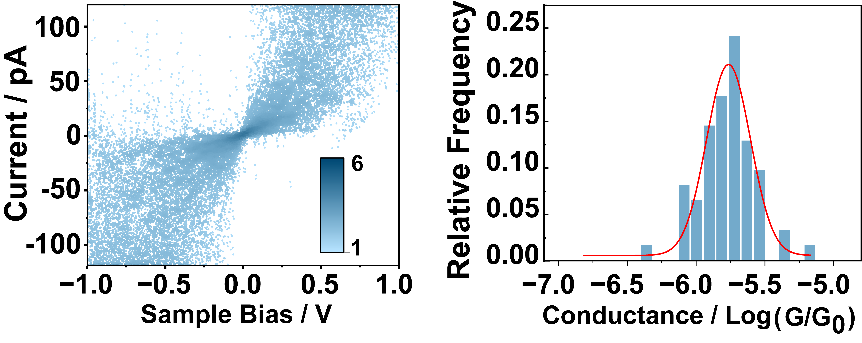
**

**Figure S20.** The intensity map of I-V curves **(left)** recorded from thrombin bivalently anchored to DNA origami via two aptamers in a nitrogen glovebox (n = 62), and corresponding one-dimensional conductance histograms **(right)** obtained from linear fitting within ±0.4 V, showing a decreased average conductance of 10^-5.75^ G_0_. The color bar represents the number of counts (density) in each grid. Both voltage (x) and current (y) axes are divided into 151 grids.

**
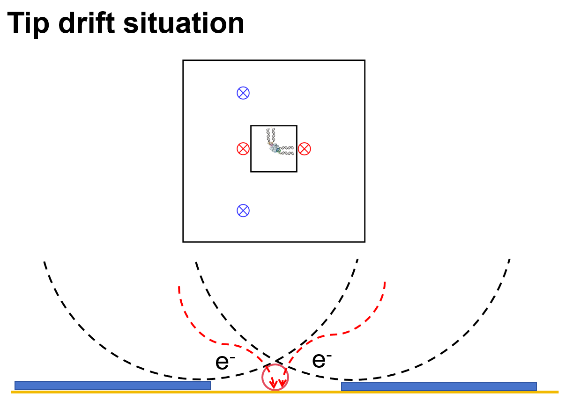

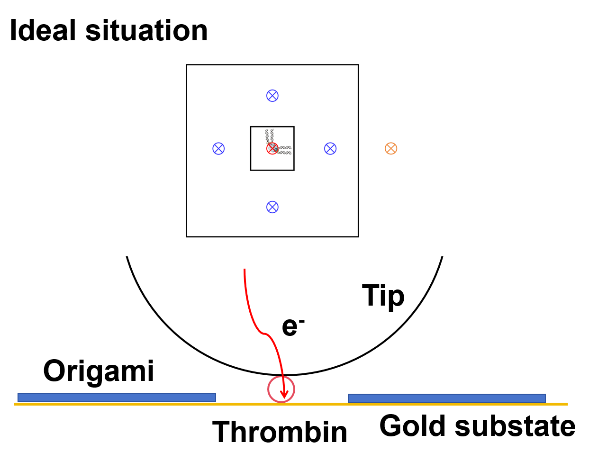
**

**Figure S21.** **Schematic illustration of contact-induced measurement errors due to tip size and drift. Left (Ideal situation):** The AFM tip contacts the center of the thrombin molecule, achieving optimal alignment for single-molecule electronic measurement. **Right (Tip drift situation):** Tip drift causes misalignment, resulting in contact at the periphery of the protein and partial overlap with the DNA origami. This misalignment introduces variability in contact geometry, leading to increased measurement uncertainty.
 These errors stem from two main factors: the relatively large curvature radius of the conductive probe (~25 nm) compared to the size of the thrombin molecule (~3.5 nm) and the DNA origami’s central cavity (~20 nm), and the drift in tip position between imaging and measurement steps.


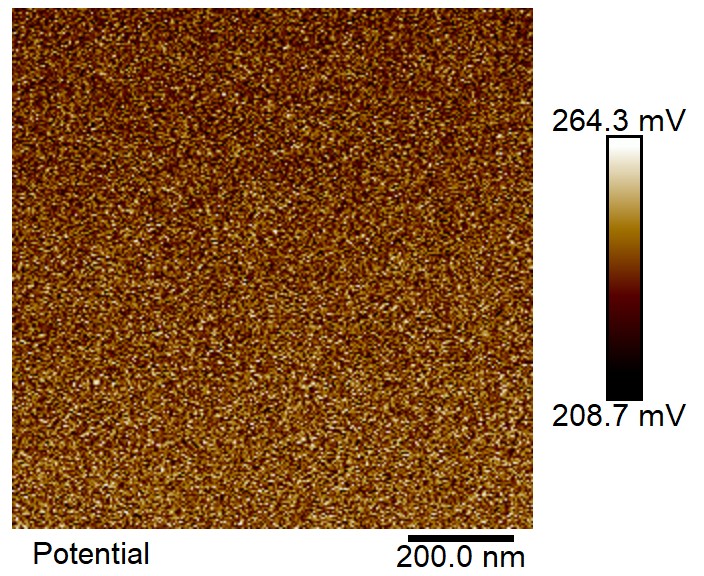

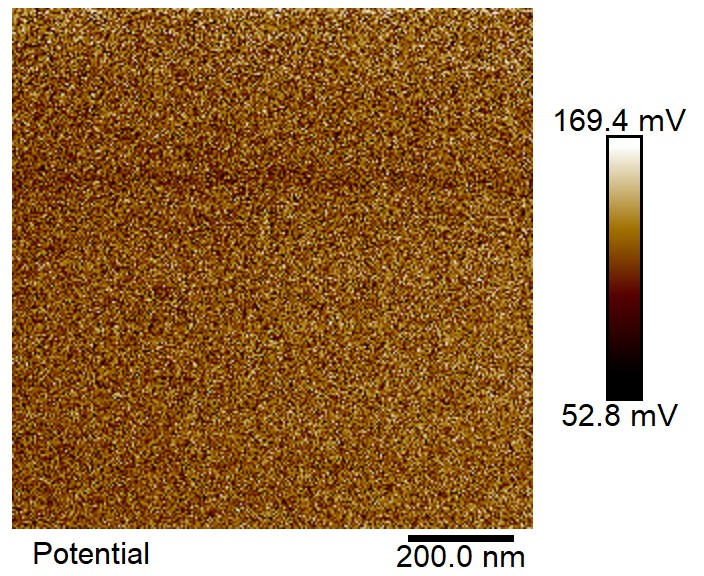
**Figure S22.** Surface potential mapping of the HOPG interface using different conductive probes. Kelvin probe force microscopy (KPFM) measurements of highly ordered pyrolytic graphite (HOPG) using a Pt-Ir-coated probe SCM-PIV-V2 (**left**, average surface potential: 0.117 V) and an Au-coated probe MULTI75GB (**right**, average surface potential: 0.237 V).

**DNA origami sequence**

**
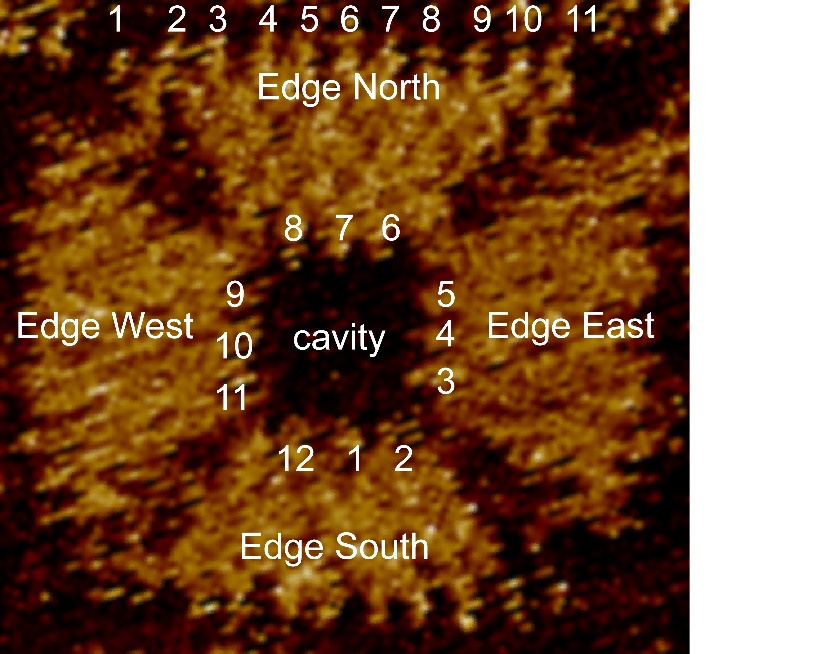
**

**Table S1.** Thiolated DNA(3’-C_3_-S-S-C_3_).

| **Name** | **Sequence（5' to 3'）** |
| --- | --- |
| Edge W2 | GTGTCGTAGACACCAAAGACACCACGGAAACATATAAAAGAAACGTTTTT |
| Edge W4 | GTGTCGTAGACACCGCGTTTTCATCGGCATAGCGTCAGACTGTAGTTTTT |
| Edge W6 | GTGTCGTAGACACCCGCCACCCTCAGAGCGCCGCCACCCTCAGAATTTTT |
| Edge W8 | GTGTCGTAGACACTAAATATGCAACTAAAGTAGCTCAACATGTTTTTTTT |
| Edge W10 | GTGTCGTAGACACGCGGGAGAAGCCTTTAACCCTGTAATACTTTTTTTTT |
| Edge S2 | GTGTCGTAGACACGTCAATCATATGTACCATCGTAAAACTAGCATTTTTT |
| Edge S4 | GTGTCGTAGACACGCATCTGCCAGTTTGAGGCGCATCGTAACCGTTTTTT |
| Edge S6 | GTGTCGTAGACACCAACTGTTGGGAAGGGCGCCATTCAGGCTGCGTTTTT |
| Edge S8 | GTGTCGTAGACACACGACGGCCAGTGCCAGTCACGACGTTGTAAATTTTT |
| Edge S10 | GTGTCGTAGACACGGTTTTTCTTTTCACCTATTGGGCGCCAGGGTTTTTT |
| Edge E2 | GTGTCGTAGACACTGAAAGCGTAAGAATATAGAACCCTTCTGACCTTTTT |
| Edge E4 | GTGTCGTAGACACGGAGCACTAACAACTAATCTAAAATATCTTTATTTTT |
| Edge E6 | GTGTCGTAGACACTAAAAGTTTGAGTAACCGAACGTTATTAATTTTTTTT |
| Edge E8 | GTGTCGTAGACACATACTTCTGAATAATGCCTGATTGTTTGGATTTTTTT |
| Edge E10 | GTGTCGTAGACACAGTACATAAATCAATACTTTTTTAATGGAAACTTTTT |
| Edge N2 | GTGTCGTAGACACTCTTCTGACCTAAATTATTTTAGTTAATTTCATTTTT |
| Edge N4 | GTGTCGTAGACACTGCAGAACGCGCCTGTCAACATGTTCAGCTAATTTTT |
| Edge N6 | GTGTCGTAGACACCGGGTATTAAACCAAGCTTATCATTCCAAGAATTTTT |
| Edge N8 | GTGTCGTAGACACGAACGCGAGGCGTTTTTTATCCGGTATTCTAATTTTT |
| Edge N10 | GTGTCGTAGACACAGGGTAATTGAGCGCTCCCTGAACAAAGTCAGTTTTT |

**Table S2.** Aptamer sequence.

***Thrombin aptamer Sequence***

20-HD22 binding with Bms cavity 1, 20-TBA15 binding with Bms cavity 4.

| 20-HD22 | TGAATCTAACGGTGCGTATC-TTTTAGTCCGTGGTAGGGCAGGTTGGGGTGACTTTTTGTACCTCGGAGACCAAGTAG |
| --- | --- |
| 20-TBA15 | CATCTGACCTCTGTGCTGCT-TTTTGGTTGGTGTGGTTGGTTTTTTCACGGTAGCACGCATAGG |

***SA aptamer Sequence***

20-SAA1 binding with Bms cavity 2,3,5,6.

| 20-SAA1 | TGAATCTAACGGTGCGTATCTTTTTCTGTGAGACGACGCACCGTCGCAGGTTTTGTCTCACAGTTTTGTACCTCGGAGACCAAGTAG |
| --- | --- |

**Table S3.** Unmodified DNA sequence.

| **Name** | **Sequence（5' to 3'）** |
| --- | --- |
| ums 1 | TTCAACCGTTCTAGCTGGAGAGAT |
| ums 2 | AAAAACGCTCATGGAAAGGATTAT |
| ums 3 | CATAGGTCTGAGAGACTATAACTA |
| ums 4 | ACCAGAAGGAAACCGAGCTGGCAT |
| ums 5 | CTACAAAGAATCGATGAACGGTA |
| ums 6 | TTACATTGTCTGGCCAACAGAGA |
| ums 7 | TATGTAAAAACTTTTTCAAATAT |
| ums 8 | GATTAAGATACATAAAGGTGGCA |
| ums 9 | AACATGAGCTCAGTACCAGGCGTAGCCCGG |
| ums 10 | CGCCATTCGATCGGTGCATAAAAACCAAAA |
| ums 11 | TATTCATTGGCTTTTGCAAAAGAAGAGCAACA |
| ums 12 | CGGAATCGAATGACCATAAATCCAGAAGCA |
| ums 13 | CTTTGCCATTATCATTTCTGCTCCATGTTA |
| ums 14 | TAGGCTGGGAACGAGGCGCAGACGGTATCATC |
| ums 15 | TACAGACATTCATTACCCAAATGGCTTGCC |
| ums 16 | GTCTTTCTACCGCACTCAATAGAAAGGAAC |
| ums 17 | TGCTTTCGGAATTGCGAATAATAATTTGCTAA |
| ums 18 | ATCGGTTCCGACAATGACAACACGCTGAGG |
| ums 19 | CCTCAGACACCACCCTCGGTAATAAGTTTT |
| ums 20 | AGAGGCTGCAGTGCCTTGAGTAACGCGTCATA |
| ums 21 | TAGCGAGAGAATCCCCCTCAAACCAGGCAAAG |
| ums 22 | CTTAGCCGCTGACCTTCATCTCGTATTAAATC |
| ums 23 | AACTAAAGAGGTGAATTTCTCAATAATCGGCT |
| ums 24 | AACGGGGTAGACTCCTCAACCGGAACCGCCTC |
| ums 25 | CCAGTAAAGTGCCCGTATAAACTATTCTGA |
| ums 26 | TTACCAGACGACGGGGCCTCTTCCTGCAAG |
| ums 27 | CTATCATATCATCAGTTGAGATTTACGAACTA |
| ums 28 | ATAGTAAGTTTTGCCAGAGGGGCAATACTG |
| ums 29 | CGAAATCCGCGACTGCGGAACAATATTCCT |
| ums 30 | GCCTGATAGCAAAAGAATACACTATAATGCCA |
| ums 31 | GAGATTTGTCAATCATAAGGGAGAACGGTG |
| ums 32 | TCAGCGGAGTGAGATCGAGAACAATTACCG |
| ums 33 | ACAACTTTCAGCCCTCATAGTTAGCACCAGTA |
| ums 34 | ATGGGATTTTTTTCACGTTGAATTAATTGT |
| ums 35 | ACAGGAGTGTACTAGAGCCGCCAGACAGGA |
| ums 36 | CATGGCTTCCTTGATATTCACAAAGGTCATTTTTGCGGAT |
| ums 37 | GCGATTAAGTTGGGGTAGAAAGATACCCTCGT |
| ums 38 | GATTATCAGATGAAAACGAAAGAGAATTGTGT |
| ums 39 | CGCCCAATAGCAACATTCCACAGACAACAGTT |
| ums 40 | GGTTGAGGGCTGAATATAATGCTGTACGGT |
| ums 41 | GATAAGACAAATAAATCCTCATTTACCGTT |
| ums 42 | TTTCCCAAGCTTGCATGCCTGCGGAAGCAT |
| ums 43 | ACGGAACAATCCCCGGGTACCGAGATCCGCTC |
| ums 44 | TAATAAAAGGAATACCACATTCTACGAGGC |
| ums 45 | ATATAATGAAGGGTTAGAACCTATAACGGA |
| ums 46 | CTACGAAGTTGCACGTAAAACAGACAGTAACA |
| ums 47 | AAATACGAAACACTCATCTTTGGTACAACG |
| ums 48 | AGAAGGCAGCGAACCTCCCGACGCCAGTTA |
| ums 49 | CAAACTACAGCCTTAAATCAAGATACGCTAAC |
| ums 50 | GTTTCGTCGTAACGATCTAAAGTTTTCTGT |
| ums 51 | GGCTTAGAGCTTAATTCAGGTCAGACGATTGGTTGATGAT |
| ums 52 | TCTAGAGGACATTATTACAGTAACGCCAGGGT |
| ums 53 | AAAATTATGCACCAACCTATGGCAATTCATCA |
| ums 54 | GGTTTTGAAACGCCTGTAGGCAAATCAGATAT |
| ums 55 | GTCTGGAAGGGGCGCGAGCTGAAATTAACA |
| ums 56 | CCATATAACAGTTGATTCCCAATTGGTCAATA |
| ums 57 | CGCAAATCTGCGAACGAGTAGACTCCTTTT |
| ums 58 | AAAGTGTCACATTAATTGCGTTGAGAGGCG |
| ums 59 | AATTGTTCTCGAATTCGTAATCATCTACGT |
| ums 60 | TTCGCCTCAGAGGCGAATTATTTTTCATTT |
| ums 61 | GAATATAAATAAAGAAATTGCGAACGGGTA |
| ums 62 | CAAAATAGAAACGATTTTTTGTAGAATTAA |
| ums 63 | CTTACCATAGTTGCTATTTTGCAACACTGA |
| ums 64 | TGCCCGCTACACAACATACGAGCCAGGTCGAC |
| ums 65 | TTACCTGATACATCGGGAGAAACAACCATATC |
| ums 66 | AAAAATGATTCCAGAGCCTAATTTTTGCGGGA |
| ums 67 | TCCAATAAACCAAAAACATTATGTTTCAAC |
| ums 68 | GGCAAGGCAGCTATATTTTCATTTGTTTCATT |
| ums 69 | GTTTGCGAGTGAGACGGGCAACGCAAGCGG |
| ums 70 | TCGGCCAACGCGCGGGGCGCTCAC |
| ums 71 | GAATTACTATGTGAGTGAATAATAGAATCC |
| ums 72 | TAATTACATTTAACAACATTTCAA |
| ums 73 | CTGAACAAATATCAGAGAGATAAACAATGA |
| ums 74 | AAGCGCATTAGACGGGTTAACGTC |
| ums 75 | AAGCTAAATCGGTTGTATCATACA |
| ums 76 | GCAAGGATTGAGTAATGTGTAGGCAGTCAAATCACCATCA |
| ums 77 | TCCACGCATGGTGGTTCCGCCATT |
| ums 78 | TTGAAAAGAGTCAATAGTGAATTT |
| ums 79 | AATAGCAAGTAAGCAGATAGCCGA |
| ums 80 | CCGGTTGATAATCAGGCATTAAA |
| ums 81 | CGTGGCACAGACAATGAACCACC |
| ums 82 | TAATGGTTTGAAATAAGTATCAT |
| ums 83 | TAAGTTTATTTTGTCGGAAGGTA |
| ums 84 | TTTTTGTCCATCAAAAATAATTACGTTGGT |
| ums 85 | AACGTTAAGTAGCCAGCTTTCATCCAAACGGC |
| ums 86 | AGCAGAAACCGCCTGCAACAGTGAATTGAG |
| ums 87 | ACATCGCCGCAGCAAATGAAAAATATCTGGTC |
| ums 88 | ATGCGTTCAACAGTAGGGCTTAGACGACGA |
| ums 89 | ATAATTACTTTAACAACGCCAACAAGTACCGA |
| ums 90 | AATATTGCGTCACCGACTTGAGAATCAAGT |
| ums 91 | GCGACATTGCCAGCAAAATCACCAATCGATAG |
| ums 92 | GCCTTCCTTATTTTGTTAAAATTCAAAAGCCC |
| ums 93 | GAGAGCCAATTAAAAATACCGAACATTTTTGA |
| ums 94 | TCGCCATATAGAAAAAGCCTGTTTCCGACCGT |
| ums 95 | GAATTAGACAACCGATTGAGGGAGACAATCAA |
| ums 96 | GTAGATGGGGGACGACGCAAATATCGCGTT |
| ums 97 | GGATTGACAGCTTCAAAGCGAACCTTGCATCA |
| ums 98 | GAAGGTTATAGATTAGAGTTTAATTTCAAC |
| ums 99 | AGTTGGCATTGTGAATTACCTTATGAAACACC |
| ums100 | CAATAAATTATCAACAAGCAGCGAAAGACA |
| ums 101 | CAAAAGGTACGAGGGTAGCAACGGGGAGTTAA |
| ums 102 | TTGCCTTTTTTCGGTCACAGAACCGCCACC |
| ums 103 | CAGCACCGCGCCACCCTCAGAGCCGTATCACC |
| ums 104 | TTAATTCGCGTAATGGGATAGGTCCGCGTCTG |
| ums 105 | TTTAATCAAATCAACAGTTGAAAGGCCACGCT |
| ums 106 | GCATCGGAAAAGTAATTCTGTCCAATTGAGAA |
| ums 107 | CTCAGAACTAATCAGTAGCGACAGCCATTTGG |
| ums 108 | AATAGGTACCACCCTCATTTTCGTCACCAA |
| ums 109 | GCCCGAAAGACTTACAGTATCGGGGCACCG |
| ums 110 | AAAAGATTACAGTTCAGAAAACGAGTCATAAA |
| ums 111 | AAGCGGAAGACCGGAAGCAAACGATTCTCC |
| ums 112 | TGGGCTTGAGATGGCCGTCAATACTTTACA |
| ums 113 | AGAACGAGTTGACAAGAACCGGATCAGGCGCA |
| ums 114 | CTGACGAGCGATTTTAAGAACTAAACCCTC |
| ums 115 | ATCGTCACCCTCATAGATAAGTCTACGAGC |
| ums 116 | AGGCCGCTGATACCGATAGTTGCGTATCAGCT |
| ums 117 | CTTGCAGCTACAGAGGCTTTGAAATAAGAG |
| ums 118 | GTACCGCCACCCTTAGCCCCCTTCACCGGA |
| ums 119 | GTACTCAGGGATTAGCGGGGTTTTAAGTATTA |
| ums 120 | CTTCTGGTGCCGGAAATGCTTTAAAAGAGGAA |
| ums 121 | AACAATTCGACAACAAGAGTAATCTAGTAAAT |
| ums 122 | ATGTAGAAACCAATTAAACAGCTTTTTGCGGG |
| ums 123 | ACCAGAGCCACCAGAGAAGGATTAGAGGTTTA |
| Edge W1 | GTGTCGTAGACACGGAATACCCAAAAGAAGAAACGCAATAATAACGTGTCGTAGACAC |
| Edge W2 | GTGTCGTAGACACCAAAGACACCACGGAAACATATAAAAGAAACGGTGTCGTAGACAC |
| Edge W3 | GTGTCGTAGACACAAAGGTGAATTATCACACGGAAATTATTCATTGTGTCGTAGACAC |
| Edge W4 | GTGTCGTAGACACCGCGTTTTCATCGGCATAGCGTCAGACTGTAGGTGTCGTAGACAC |
| Edge W5 | GTGTCGTAGACACTTTTCATAATCAAAATATTAGCGTTTGCCATCGTGTCGTAGACAC |
| Edge W6 | GTGTCGTAGACACCCGCCACCCTCAGAGCGCCGCCACCCTCAGAAGTGTCGTAGACAC |
| Edge W7 | GTGTCGTAGACACAGCCGCCGCCAGCATTCCAGAACCACCACCAGGTGTCGTAGACAC |
| Edge W8 | GTGTCGTAGACACTAAATATGCAACTAAAGTAGCTCAACATGTTTGTGTCGTAGACAC |
| Edge W9 | GTGTCGTAGACACTACTAATAGTAGTAGCAAGGTGGCATCAATTCGTGTCGTAGACAC |
| Edge W10 | GTGTCGTAGACACGCGGGAGAAGCCTTTAACCCTGTAATACTTTTGTGTCGTAGACAC |
| Edge W11 | GTGTCGTAGACACTGAGAAAGGCCGGAGATAAAGATTCAAAAGGGGTGTCGTAGACAC |
| Edge S1 | GTGTCGTAGACACGAGGGTAGCTATTTTTATAAATTAATGCCGGAGTGTCGTAGACAC |
| Edge S2 | GTGTCGTAGACACGTCAATCATATGTACCATCGTAAAACTAGCATGTGTCGTAGACAC |
| Edge S3 | GTGTCGTAGACACTTAACCAATAGGAACGTAAATCAGCTCATTTTGTGTCGTAGACAC |
| Edge S4 | GTGTCGTAGACACGCATCTGCCAGTTTGAGGCGCATCGTAACCGTGTGTCGTAGACAC |
| Edge S5 | GTGTCGTAGACACCTCCAGCCAGCTTTCCCCTCAGGAAGATCGCAGTGTCGTAGACAC |
| Edge S6 | GTGTCGTAGACACCAACTGTTGGGAAGGGCGCCATTCAGGCTGCGGTGTCGTAGACAC |
| Edge S7 | GTGTCGTAGACACGCGAAAGGGGGATGTGGCTATTACGCCAGCTGGTGTCGTAGACAC |
| Edge S8 | GTGTCGTAGACACACGACGGCCAGTGCCAGTCACGACGTTGTAAAGTGTCGTAGACAC |
| Edge S9 | GTGTCGTAGACACAATGAGTGAGCTAACTAAAGCCTGGGGTGCCTGTGTCGTAGACAC |
| Edge S10 | GTGTCGTAGACACGGTTTTTCTTTTCACCTATTGGGCGCCAGGGTGTGTCGTAGACAC |
| Edge S11 | GTGTCGTAGACACGCGAAAATCCTGTTTGTGGTTTGCCCCAGCAGGTGTCGTAGACAC |
| Edge E1 | GTGTCGTAGACACCTCAATCGTCTGAAATTACCTACATTTTGACGGTGTCGTAGACAC |
| Edge E2 | GTGTCGTAGACACTGAAAGCGTAAGAATATAGAACCCTTCTGACCGTGTCGTAGACAC |
| Edge E3 | GTGTCGTAGACACGGCGGTCAGTATTAACGATAAAACAGAGGTGAGTGTCGTAGACAC |
| Edge E4 | GTGTCGTAGACACGGAGCACTAACAACTAATCTAAAATATCTTTAGTGTCGTAGACAC |
| Edge E5 | GTGTCGTAGACACATTTAGAAGTATTAGAGATAATACATTTGAGGGTGTCGTAGACAC |
| Edge E6 | GTGTCGTAGACACTAAAAGTTTGAGTAACCGAACGTTATTAATTTGTGTCGTAGACAC |
| Edge E7 | GTGTCGTAGACACAGCGGAATTATCATCAAGAAACCACCAGAAGGGTGTCGTAGACAC |
| Edge E8 | GTGTCGTAGACACATACTTCTGAATAATGCCTGATTGTTTGGATTGTGTCGTAGACAC |
| Edge E9 | GTGTCGTAGACACAAGTTACAAAATCGCGGATTGCTTTGAATACCGTGTCGTAGACAC |
| Edge E10 | GTGTCGTAGACACAGTACATAAATCAATACTTTTTTAATGGAAACGTGTCGTAGACAC |
| Edge E11 | GTGTCGTAGACACATTAAGACGCTGAGAACATAGCGATAGCTTAGGTGTCGTAGACAC |
| Edge N1 | GTGTCGTAGACACGCTTAGGTTGGGTTATACCTTTTTAACCTCCGGTGTCGTAGACAC |
| Edge N2 | GTGTCGTAGACACTCTTCTGACCTAAATTATTTTAGTTAATTTCAGTGTCGTAGACAC |
| Edge N3 | GTGTCGTAGACACGTATAAAGCCAACGCTATACAAATTCTTACCAGTGTCGTAGACAC |
| Edge N4 | GTGTCGTAGACACTGCAGAACGCGCCTGTCAACATGTTCAGCTAAGTGTCGTAGACAC |
| Edge N5 | GTGTCGTAGACACATATCCCATCCTAATTCTGAACAAGAAAAATAGTGTCGTAGACAC |
| Edge N6 | GTGTCGTAGACACCGGGTATTAAACCAAGCTTATCATTCCAAGAAGTGTCGTAGACAC |
| Edge N7 | GTGTCGTAGACACTTTCATCGTAGGAATCAGCAAGCCGTTTTTATGTGTCGTAGACAC |
| Edge N8 | GTGTCGTAGACACGAACGCGAGGCGTTTTTTATCCGGTATTCTAAGTGTCGTAGACAC |
| Edge N9 | GTGTCGTAGACACATCCCAATCCAAATAAAACAGCCATATTATTTGTGTCGTAGACAC |
| Edge N10 | GTGTCGTAGACACAGGGTAATTGAGCGCTCCCTGAACAAAGTCAGGTGTCGTAGACAC |
| Edge N11 | GTGTCGTAGACACAGCCCTTTTTAAGAAAATAGCTATCTTACCGAGTGTCGTAGACAC |
| connection 1 | AGAGTACCTTTAATTGTTTAGTTTTTCCCGTCGTCCAACAGGTCAGGAT |
| connection 2 | GACGTTGGGAAGAAAAATGGTCATTTAAATATCGGCTCATTATACCAGT |
| connection 3 | GAGGAAGTTTCCATTATAGATTTTTTAGCCAGTGGACTAAAGACTTTTT |
| connection 4 | GGAACCCATGTACCGTACCCAGCTTTCGGAAACAGGGATAGCAAGCCCA |
| connection 5 | ACAATTCCTTCCAGTCGGGAAACCTGTCGTTTTTTTTGATAGCCCTAAA |
| connection 6 | GTACCTTTGCAAAAGAAGATGATGAAACATTTTTTTAAACACCGGAATC |
| connection 7 | GAGCGTCTAAATAGCAGCCTTTACAGAGATTTTTTTCAAAGACAAAAGG |
| connection 8 | ACCTGTTTAAAGAATTAGCAAAATTAAGCTTTTTTTTATTTAAATTGTA |
| connection 9 | GCCCTTCACCGTTTTTTTCCAGTAATAAA |
| connection 10 | CTGTAAATCGTTTTTTTTAAGACAAAGAA |
| connection 11 | GAATTGAGTTATTTTTTTAGCAAACGTAG |
| connection 12 | TTAGAACCCTCTTTTTTTAGTCTGGAGCA |
| connection 13 | AACAAGAGGCTATCAGGTTTTTTTTTATGCAATGCCAAAAATTT |
| connection 14 | AGGGACATGCAGATTCACTTTTTTTTGAGAGTTGCAAGCTGATT |
| connection 15 | CGCGAGAATGCTGATGCATTTTTTTTAATTTTCCCTCCTTGCTT |
| connection 16 | AAAATACACTCCTTATTATTTTTTTTAAGAGCAAGAACCCACAA |
| connection 17 | CAAAAACAGGAAGATTGTATTTCCTCAGAGCATA |
| connection 18 | ATGGCTATTAGTCTTTAATGTTTGCATTAATGAA |
| connection 19 | GTGATAAATAAGGCGTTAAATTAGAAAACAAAAT |
| connection 20 | TAGAAAATTCATATGGTTTATTATAAAAACAGGG |
| connection 21 | TGAAACCGTAGCACCATTACCTTTTTTCCTGAAT |
| connection 22 | GTGGGAAAACATTAAATGTGATTTTTTTACATTT |
| connection 23 | AATCAATCTAAAGCATCACCTTTTTTTTGTGTGA |
| connection 24 | AATATAATGTAATTTAGGCAGTTTTTTGTCAGAT |
| ums cavity 1 | GTGTCGTAGACACTAGACTGGATAGCGTCGTAATAGTAAAATGTTGTGTCGTAGACAC |
| ums cavity 2 | GTGTCGTAGACACTAACGCCAAAAGGAATAACTAATGCAGATACAGTGTCGTAGACAC |
| ums cavity 3 | GTGTCGTAGACACCTCATTCAGTGAATAACAACGTAACAAAGCTGGTGTCGTAGACAC |
| ums cavity 4 | GTGTCGTAGACACTTGAAAGAGGACAGATACCGAACTGACCAACTGTGTCGTAGACAC |
| ums cavity 5 | GTGTCGTAGACACCCAAGCGCGAAACAAAACCCCCAGCGATTATAGTGTCGTAGACAC |
| ums cavity 6 | GTGTCGTAGACACAACCGATATATTCGGTACCATCGCCCACGCATGTGTCGTAGACAC |
| ums cavity 7 | GTGTCGTAGACACGCTCCAAAAGGAGCCTAATCTCCAAAAAAAAGGTGTCGTAGACAC |
| ums cavity 8 | GTGTCGTAGACACGACGTTAGTAAATGAATTTTGTCGTCTTTCCAGTGTCGTAGACAC |
| ums cavity 9 | GTGTCGTAGACACAGGGTTGATATAAGTAGATAAGTGCCGTCGAGGTGTCGTAGACAC |
| ums cavity 10 | GTGTCGTAGACACCTATTTCGGAACCTATAGTTAATGCCCCCTGCGTGTCGTAGACAC |
| ums cavity 11 | GTGTCGTAGACACAGCGCAGTCTCTGAATTAAAGCCAGAATGGAAGTGTCGTAGACAC |
| ums cavity 12 | GTGTCGTAGACACCCCTGACTATTATAGTAAAAATCAGGTCTTTAGTGTCGTAGACAC |

**Table S4.** Thrombin cavity and arm sequence.

| Bms cavity 1 | GATACGCACCGTTAGATTCATAGACTGGATAGCGTCGTAATAGTAAAATGTTCTACTTGGTCTCCGAGGTAC |
| --- | --- |
| Bms cavity 4 | GATACGCACCGTTAGATTCATTGAAAGAGGACAGATACCGAACTGACCAACTCTACTTGGTCTCCGAGGTAC |

**Table S5.** SA cavity and arm sequence.

| Bms cavity 2 | GATACGCACCGTTAGATTCATAACGCCAAAAGGAATAACTAATGCAGATACACTACTTGGTCTCCGAGGTAC |
| --- | --- |
| Bms cavity 3 | GATACGCACCGTTAGATTCACTCATTCAGTGAATAACAACGTAACAAAGCTGCTACTTGGTCTCCGAGGTAC |
| Bms cavity 5 | GATACGCACCGTTAGATTCACCAAGCGCGAAACAAAACCCCCAGCGATTATACTACTTGGTCTCCGAGGTAC |
| Bms cavity 6 | GATACGCACCGTTAGATTCAAACCGATATATTCGGTACCATCGCCCACGCATCTACTTGGTCTCCGAGGTAC |

**Supplementary References**

[1] J. A. Bittker, B. V. Le, D. R. Liu. "Nucleic acid evolution and minimization by nonhomologous random recombination," Nature Biotechnology, **2002**, 20, 1024. https://doi.org/10.1038/nbt736

[2] A. O. Pineda, C. J. Carrell, L. A. Bush, S. Prasad, S. Caccia, Z.-W. Chen, F. S. Mathews, E. Di Cera. "Molecular Dissection of Na+ Binding to Thrombin*," Journal of Biological Chemistry, 2004, 279, 31842. https://doi.org/10.1074/jbc.M401756200

[3] I. M. Kovach. " Proton Bridging in Catalysis by and Inhibition of Serine Proteases of the Blood Cascade System," *Life*, **2021**, 11. https://doi.org/10.3390/life11050396
